# Conservation status and historical relatedness of South African communal indigenous goat populations using a genome-wide single-nucleotide polymorphism marker

**DOI:** 10.3389/fgene.2022.909472

**Published:** 2022-08-09

**Authors:** T. C. Chokoe, K. Hadebe, F. C. Muchadeyi, K. A. Nephawe, E. F. Dzomba, T. D. Mphahlele, T. C. Matelele, B. J. Mtileni

**Affiliations:** ^1^ Farm Animal Genetic Resources, Department of Agriculture, Land Reform and Rural Development, Pretoria, South Africa; ^2^ School of Agriculture & Environmental Sciences, University of Limpopo, Polokwane, South Africa; ^3^ Biotechnology Platform, Agricultural Research Council, Pretoria, South Africa; ^4^ Department of Animal Sciences, Tshwane University of Technology, Pretoria, South Africa; ^5^ Discipline of Genetics, School of Life Sciences, University of Kwazulu-Natal, Scottsville, South African

**Keywords:** runs of homozygosity, effective population size, populations, genomic inbreeding, conservation

## Abstract

Indigenous goats form the majority of populations in smallholder, low input, low output production systems and are considered an important genetic resource due to their adaptability to different production environments and support of communal farming. Effective population size (*N*_*e*_), inbreeding levels, and the runs of homozygosity (ROHs) are effective tools for exploring the genetic diversity and understanding the demographic history in efforts to support breeding strategies to use and conserve genetic resources. Across populations, the current *N*_*e*_ of Gauteng was the lowest at 371 animals, while the historical *N*_*e*_ across populations suggests that the ancestor *N*_*e*_ has decreased by 53.86%, 44.58%, 42.16%, and 41.16% in Free State (FS), North West (NW), Limpopo (LP), and Gauteng (GP), respectively, over the last 971 generations. Genomic inbreeding levels related to ancient kinship (*F*_*ROH*_ > 5 Mb) were highest in FS (0.08 ± 0.09) and lowest in the Eastern Cape (EC) (0.02 ± 0.02). A total of 871 ROH island regions which include important environmental adaptation and hermo-tolerance genes such as *IL10RB, IL23A, FGF9, IGF1, EGR1, MTOR,* and *MAPK3* were identified (occurring in over 20% of the samples) in FS (*n* = 37), GP (*n* = 42), and NW (*n* = 2) populations only. The mean length of ROH across populations was 7.76 Mb and ranged from 1.61 Mb in KwaZulu-Natal (KZN) to 98.05 Mb (GP and NW). The distribution of ROH according to their size showed that the majority (*n* = 1949) of the detected ROH were > 5 Mb in length compared to the other categories. Assuming two hypothetical ancestral populations, the populations from KZN and LP are revealed, supporting PC 1. The genomes of KZN and LP share a common origin but have substantial admixture from the EC and NW populations. The findings revealed that the occurrence of high *N*_*e*_ and autozygosity varied largely across breeds in communal indigenous goat populations at recent and ancient events when a genome-wide single-nucleotide polymorphism (SNP) marker was used. The use of Illumina goat SNP50K BeadChip shows that there was a migration route of communal indigenous goat populations from the northern part (LP) of South Africa to the eastern areas of the KZN that confirmed their historical relatedness and coincides with the migration periods of the Bantu nation.

## Introduction

There are currently 15 South African goat genetic resources listed on the DAD-IS of FAO and 13 on the Domestic Animal Genetic Resources Information System (DAGRIS) of the International Livestock Research Institute, including those listed in DAD-IS. In the country, indigenous goat ecotypes have been used as triple-purpose animals (e.g., skin, milk, and meat); and depending on the region, the animal characteristics, and the geographical isolation, they have begun to diverge into breeds/populations (Visser et al., 2016). These ecotypes have generally been named after their place of origin (e.g., Northern Cape Skilder) and/or their prominent characteristics (e.g., Xhosa lobbed ear) and on the basis of the people who own them (e.g., Nguni) (Morrison, 2007). These ecotypes are widely spread across all major agro-ecological regions of South Africa, displaying adaptability traits to a specific habitat or production environment and represent a significant resource to satisfy present and future demands for sustainable farming in a changing environment.

Improvement of indigenous livestock has been practiced through the introduction of high-performing breeds (exotic and improved breeds), and as a result of indiscriminate mating and breeding, the majority of communal indigenous goat populations are crossbreds (Mdladla et al., 2016a). The majority of the smallholder farmers have small herds or flocks where herd sizes could be less than five animals, with the majority of these herds being non-descript, crossbred or indigenous cattle, sheep, and goats (Mthi et al., 2017; Nyamushamba et al., 2017). The reduction in local indigenous populations suggests a need for the conservation of local genetic resources through the implementation of a national conservation strategy. Various studies conducted in the smallholder communal areas showed average flock sizes of between 1 and 120 goats (Webb and Mamabolo, 2004; Dube, 2015). Furthermore, about 70% of the goats are kept under traditional management systems where the farm structure comprises of about twenty (±20) indigenous goats (Monau et al., 2020). A detailed information on the phenotypic, genetic diversity and population structure of goats ecotype populations become important to guide conservation strategies through utilization of these populations. According to Food and Agriculture Organization of the United Nations, (2015b), conservation and characterization of animal genetic resources is critical because of their contribution to the sustainable livelihoods of rural communities that depend on them for food security. Conservation frameworks should incorporate both genetic diversity and breed merits for prioritizing breeds/populations from community to national level to support breeding programs of current populations.

In South Africa, more extensive research studies on genetic diversity analyses were done using microsatellite markers that were instrumental in providing an insight into the genetic structure and variation among South African goat populations (Kotze et al., 2004; Visser et al., 2004; Kotzé et al., 2014). (Kotzé et al., 2014) observed average heterozygosity of 63% in Kalahari Reds using 18 microsatellite markers, nine of which were used in the study by (Visser et al., 2004). Recently, microsatellites have been used to study genetic variation of the Tankwa feral goat population, which showed it to be highly divergent from the other farmed populations (Kotzé et al., 2014). In spite of their common use in most livestock diversity studies, microsatellites are often criticized for their usual location in the non-coding regions of the genome and for not being directly associated with genes that affect phenotypes. This has led to low-density microsatellites finding little application in studies of the adaptive genetic diversity of local breeds.

The completion of the first draft of the goat genome (Dong et al., 2013) made it possible for the development of high-density markers (Tosser-Klopp et al., 2014). The Illumina goat SNP50K BeadChip includes 53 347 SNPs (Tosser-Klopp et al., 2014) that have found utility in South African population genetic studies in Angora (Lashmar et al., 2016), commercial, indigenous, and village goat populations (Mdladla et al., 2017), as well as investigating genetic adaptation to environmental pressures (Mdladla et al., 2018). The use of the tool has been described in other African countries (Zvinorova, 2017; Onzima et al., 2018) and in specialized breeds (Martin et al., 2016). In South Africa, research work on the use of Illumina goat SNP50K BeadChip to determine the differences of indigenous communal goats at a point of genetic background is limited as compared to the studies where microsatellites were used. Therefore, while much work on South African commercial, indigenous, and village goat populations has focused on genetic studies and investigations on genetic adaptation, less work has focused on the conservation status and historical relatedness of communal indigenous goat populations.

The presence of the extent of an effective population size (*Ne*) is an important population genetic parameter that has recently received a great deal of research attention (Zhao et al., 2014), determining population demographic development (Deng et al., 2019) and demographic processes such as migration and admixture (Nicoloso et al., 2015), and having profound implications for understanding the architecture of the animal genome (Lashmar et al., 2016; Alvarenga et al., 2018). In addition, *Ne* is widely regarded as one of the most critical population parameters because it measures the rates of genetic drift and inbreeding as well as affects the efficacy of systematic evolutionary forces such as mutation, selection, and migration (Shin et al., 2013; Silió et al., 2013). It also helps to discover population demographic history and allows for the prediction of the behavior of genetic diversity through time. The *Ne* is estimated using the r^2^ coefficient and measures the observed range and the amount of genetic variation within a frame of population genetics (Berihulay et al., 2019). It also provides information on the degree of inbreeding of the population under consideration (Flury et al., 2010). The *Ne* determines the amount of genetic variation, genetic drift, and linkage disequilibrium (LD) in populations (Liu et al., 2017). Implementation of a national conservation strategy for FAnGR must be based on a better understanding of the degree of inbreeding of the populations, genetic variation, genetic drift, and linkage disequilibrium (LD) in those populations.

An increase in inbreeding (*F*) over generations leads to a reduction in genetic diversity (Onzima et al., 2018). Higher frequency of homozygous genotypes for deleterious alleles with a reduction in individual performance (inbreeding depression) and lower population viability (Ouborg et al., 2010). Offspring may inherit autozygotic chromosomal segments from both parents that are identical by descent (IBD) when they are inbred, i.e., segments that are derived from a common ancestor (Broman and Weber, 1999). The result is the runs of homozygosity (ROH), also known as the continuous homozygous segments in the genome. The ROHs are contiguous lengths of homozygous segments of the genome where the two haplotypes inherited from the parents are identical (Gibson et al., 2006).

The extent of ROH can be used to estimate the inbreeding coefficient (Bosse et al., 2012; Martin et al., 2016; Peripolli et al., 2018), to disclose the genetic relationships among individuals, usually estimating with high accuracy the autozygosity at an individual and/or population level (Ferenčaković et al., 2011; Ferenčaković et al., 2013). Autozygosity is the homozygous state of identical by descent (IBD) alleles, which can result from several phenomena such as genetic drift, population bottlenecks, mating of close relatives, and natural and artificial selection (Curik et al., 2014; Lashmar et al., 2016). The level of selection pressure on the populations can also be established by the use of ROH (Zhang et al., 2015). Distant from more recent inbreeding may also be distinguished by the length and frequency of ROHs, since the length of IBD segments follows an inverse exponential distribution with a mean of ½ *g* Morgans, where *g* is the number of generations from a common ancestor (Howrigan et al., 2011). Effective populations (*Ne*) and ROH have been studied in humans (Gibson et al., 2006), cattle (Ferenčaković et al., 2011; Marras et al., 2014; Mastrangelo et al., 2016), pigs (Ai et al., 2013; Shin et al., 2013), and sheep (Mastrangelo et al., 2017), but less comprehensively in other livestock species, such as goats, for designing conservation strategies, especially for the communal indigenous goats of South Africa. The objective of this study was to determine the conservation status and historical relatedness of South African communal indigenous goat populations using genome-wide SNP markers.

## Methods

### Ethics approval

Permission for the study and ethical approval were obtained from the animal research ethics committees of both the University of Limpopo and the Department of Agriculture, Land Reform and Rural Development. Furthermore, verbal consent was given by the goat owners.

### Sample collection and genotyping

**FIGURE 1:**
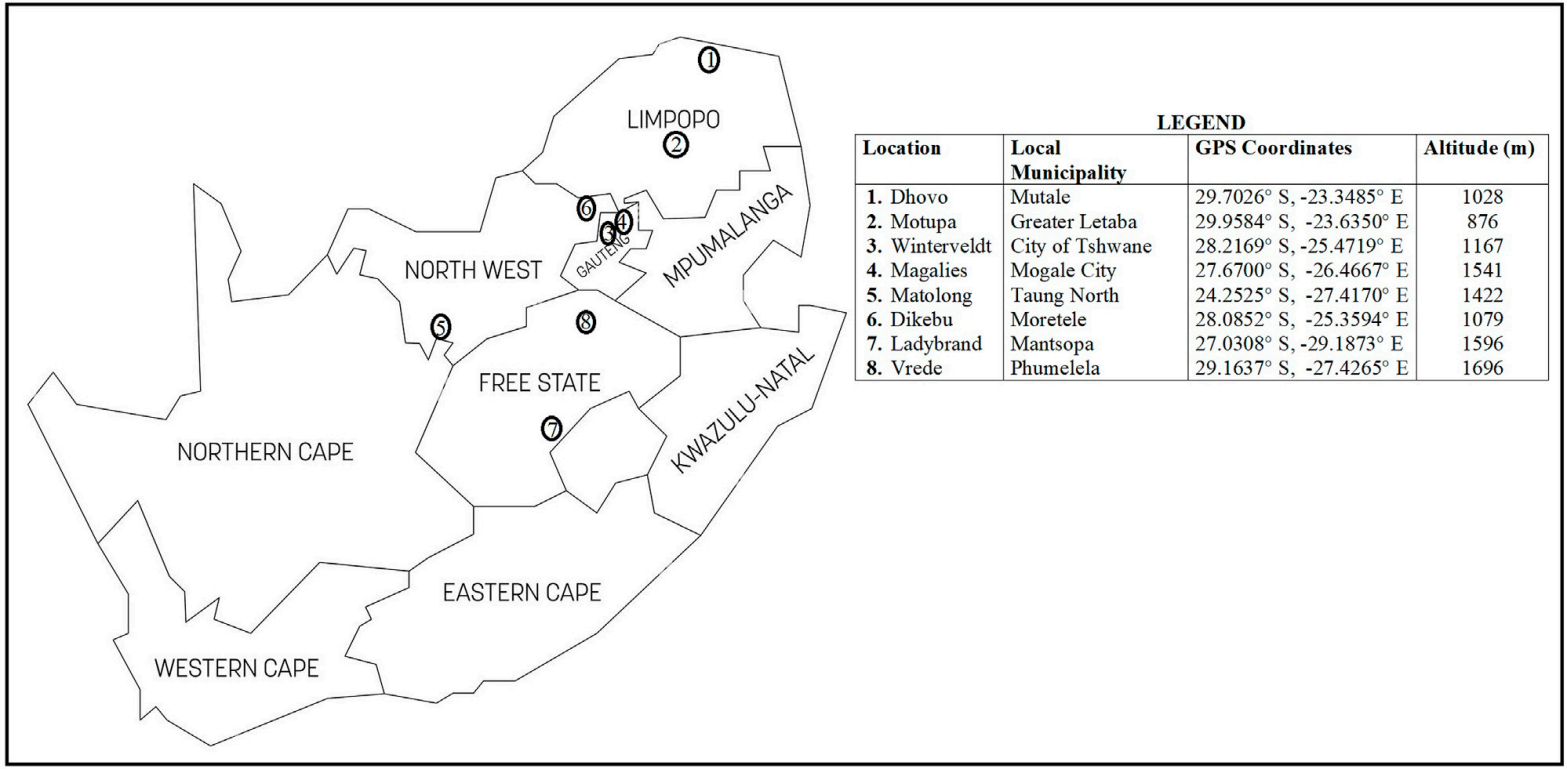
South African map showing the geographical locations of communal indigenous goat populations.

A total of 117 communal indigenous goat populations were sampled from the Free State (*n* = 24), Gauteng (*n* = 28), Limpopo (*n* = 30) and North West (*n* = 35) provinces of South Africa (Figure 1). Additionally, genotypes of communal indigenous goat populations that are under extensive production system (Mdladla, 2016) representing Eastern Cape (*n =* 20), KwaZulu-Natal (*n =* 30), Limpopo (*n =* 30) and North West (*n =* 20) provinces were included for further analysis.

All animals were genotyped using the Illumina GoatSNP50 BeadChip (Illumina Inc., San Diego, CA, United States) using the Infinium assay compatible with the Illumina HiScanSQ genotyping platform at the Agricultural Research Council—Biotechnology Platform in South Africa. A number of quality control measures were applied to all SNPs as follows: SNPs were removed if they had a call rate < 95%, a minor allele frequency (MAF) < 0.05, and if they deviated from the Hardy-Weinberg equilibrium (for a *p*-value cut-off of 0.00001), had no assigned genomic locations, and on sex chromosomes were also excluded from the analysis. The parameter “–cow” was used to indicate the number (i.e., 29) of autosomes in the goat genome since cows and goats have the same number of autosomes. The final dataset included 47,778 SNPs and 207 individuals.

### Genetic diversity indices

Historical and recent effective population sizes (*N*_*e*_) for each breed were estimated with the *SNeP* (Barbato et al., 2015), which is based on the relationship between linkage disequilibrium (LD), *N*_*e*_ and recombination rate. The different SNP marker distance bins used for r^*2*^ analysis were used to obtain different estimates of *Ne,* at different time points by calculating the number of generations (t) in the past as ½c. To verify the accuracy of the coefficient of inbreeding, the genomic coefficient was estimated *via* two methods. 1) PLINK (Purcell et al., 2007) was used to measure the inbreeding coefficient based on the difference between the observed and expected numbers of homozygous genotypes (*F*_*HOM*_) using the function –het. The calculation formula was as follows:

$$F_{HOM}=\left(E_{HOM}-O_{HOM}\right)/\left(L-E_{HOM}\right),$$

where *L* is the number of genotyped autosomal SNPs, *E*_*HOM*_ is the number of expected homozygous genotypes, and *O*_*HOM*_ is the number of observed homozygous genotypes. The inbreeding coefficient based on the proportion of autosomes covered in runs of homozygosity per individual (*F*_*ROH*_) was determined using *detectRUNs* (Biscarini et al., 2018). *F*_*ROH*_ was calculated as follows:

$$F_{ROH}=L_{ROH}/L_{AUTO},$$

where *L*_*ROH*_ is the total length of ROH on autosomes and *L*_*AUTO*_ is the total length of the autosomes covered by SNPs, which was 2450.71 Mb. Furthermore, the correlation between *F*_*ROH*_ and *F*_*HOM*_ was calculated for the four populations.

### Distribution of runs of homozygosity

Runs of homozygosity (ROH) were identified in every individual using *detectRUNS* (Biscarini et al., 2018) using a sliding window of a specified length or number of homozygous SNPs to scan along with each individual’s genotype at each SNP marker position to detect homozygous segments. The parameters and thresholds applied to define a ROH were: 1) a sliding window of 50 SNPs across the genome; 2) the minimum number of consecutive SNPs included in a ROH was 50; 3) the minimum length of a ROH was set to 1 Mb to avoid short and common ROH that occur throughout the genome due to LD (Purfield et al., 2017); and 4) a maximum of five SNPs with missing genotypes were allowed in a ROH to eliminate false positives. ROH was divided into five physical length categories (1–5 Mb, 5–10 Mb, 10–20 Mb, 20–30 Mb and < 40 Mb). The mean number of ROH per individual, the average length of ROH, the total number of ROH per animal, the percentage of chromosomes covered by ROH, and mean ROH were calculated on *detectRUNS*. The genomic inbreeding coefficient based on ROHs (*F*_*ROH*_) was also calculated as the sum of the length of the autosome covered by ROHs divided by the overall length of the autosome covered by SNPs as described by (Mastrangelo et al., 2017). The means and standard deviation (sd) of *F*_*ROH*_ were calculated as the sum of the lengths of *F*_*ROH*1–5 Mb,_ *F*_*ROH*5–10 Mb,_ *F*_*ROH*10–20 Mb or < 20 Mb._

To identify the genomic regions that were most commonly associated with ROH, the percentage of the occurrences of a SNP in ROH was calculated by counting the number of times the SNP was detected in those ROH across individuals, and this was plotted against the position of the SNP along the chromosome. This percentage threshold was normalized to 70%, 50%, and 20% of individuals per population to be an indication of a possible hotspot of ROH in the genome. The functions of these genes and pathways in which they are involved were assessed using the Kyoto Encyclopedia of Genes and Genomes (KEGG, http://www.genome.jp/kegg/) database and literature search.

### Population structure

Principal component analysis (PCA) was calculated and plotted in Golden Helix SNP and Variation Suite (SVS) V8.1 (Golden Helix, Inc. 2012). For the analysis of ancestry proportions (admixture) with K set from 2 to 10, the ADMIXTURE *v1.3.0* program (Alexander et al., 2009) was used. The default parameter of PLINK *v1.9* (50 SNPs step 5 SNPs, r^2^ 0.5) was used to subject the whole genotype data set to linkage disequilibrium (LD) pruning (Pemberton et al., 2012) prior to use in admixture analysis. Finally, to visualize admixture plots, GENESIS software was used (Buchmann and Hazelhurst, 2014).

## Results

### Genetic diversity indices

**FIGURE 2:**
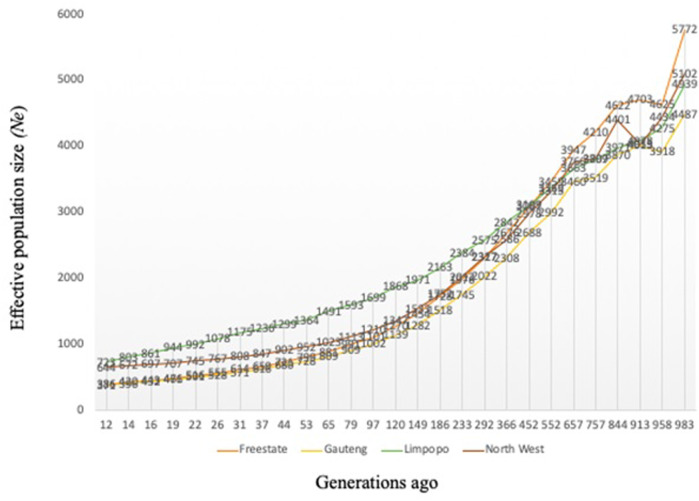
Effective population size (Ne) for the communal indigenous goat populations from Free State, Gauteng, Limpopo and North West.

Variation of the estimated *N*_*e*_ at *t* generations ago (from 12 to 983) is presented in Figure 2. As expected, *N*_*e*_ decreased progressively across generations, however, *N*_*e*_ was higher than 150 for all breeds 12 generations ago. The variation in *N*_*e*_ across generations was smallest for Gauteng (*N*_*e*_ = 371) and the Free State (*N*_*e*_ = 386), whilst Limpopo had the highest (*N*_*e*_ = 723). Ancestral populations exhibited considerably larger *N*_*e*_ values, with the largest historical *N*_*e*_ values (*N*_*e*_ = 5,772).

**FIGURE 3:**
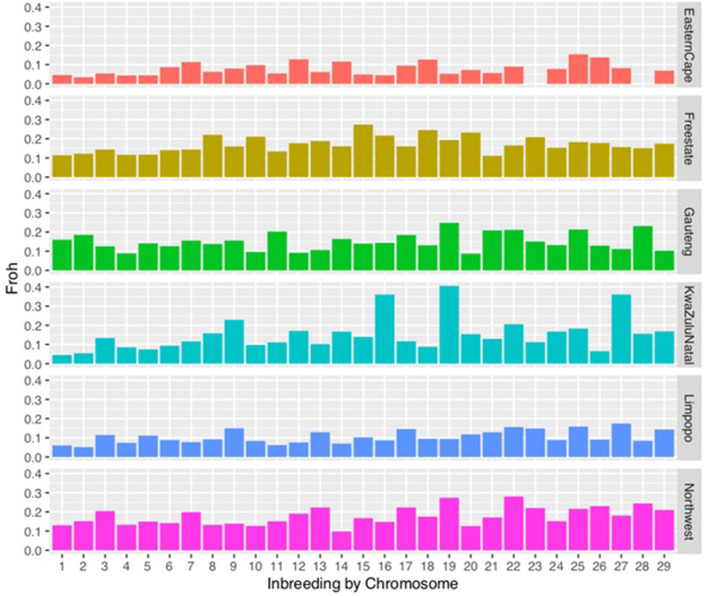
Distribution of inbreeding coefficients (FROH) based on runs of homozygosity (ROH) for each chromosome.

The average inbreeding coefficient (*F*_*IS*_) was lowest in Free State (*F*_*IS*_ = 0.03 ± 0.09), followed by 0.04 ± 0.09 in Gauteng, 0.05 ± 0.01 in the North West and the highest in Limpopo (*F*_*IS*_ = 0.09 ± 0.05). Overall, the inbreeding level was 0.06 ± 0.08. The average *F*_*ROH*_, its range of variation across populations and its distribution are summarized in Table 1. Genomic inbreeding coefficients (*F*_*ROH*_) based on the distribution of the length of runs of homozygosity by class are described in Table 2 and by chromosome in Figure 3. *F*_*ROH*_ differed significantly among populations across the length categories. The genomic inbreeding coefficients of the shortest ROH (0–5 Mb; related to ancient kinship) per animal ranged from 0.02 ± 0.02 in the Eastern Cape population to 0.08 ± 0.10 in the North West population. The *F*_*ROH*_ of Eastern Cape, Limpopo populations increased with category size, whilst decreased in Free State. Gauteng *F*_*ROH*_ decreased from 0.07 ± 0.09 to 0.05 ± 0.09 for *F*_*ROH*_ >20 Mb and increased at >20 Mb. In KwaZulu-Natal, *F*_*ROH*_ increased to up to 0.08 ± 0.09 at <20 Mb and decreased for > 20 Mb category.

**TABLE 1:** Distribution of runs of homozygosity inbreeding coefficients (*F*_*ROH*_) within each population.

Class (Mb)	Eastern cape (*n* = 20)	Free state (*n* = 24)	Gauteng (*n* = 28)	KwaZulu-natal (*n* = 25)	Limpopo (*n* = 55)	North west (*n* = 55)
0–5 Mb	0.02 ± 0.02	0.08 ± 0.09	0.07 ± 0.09	0.04 ± 0.07	0.03 ± 0.04	0.08 ± 0.10
5–10 Mb	0.02 ± 0.01	0.06 ± 0.09	0.06 ± 0.09	0.05 ± 0.08	0.03 ± 0.04	0.07 ± 0.09
10–20 Mb	0.02 ± 0.01	0.06 ± 0.08	0.05 ± 0.09	0.08 ± 0.09	0.03 ± 0.05	0.07 ± 0.09
>20 Mb	0.03 ± 0.01	0.06 ± 0.04	0.08 ± 0.12	0.06 ± 0.05	0.07 ± 0.03	0.08 ± 0.09

**TABLE 2:** Number of runs of homozygosity (*n*ROH) and length (in Mb) categorized by ROH length class (ROH_1_–_5 Mb,_ ROH_5_–_10 Mb_, ROH_10_–_20 Mb,_ ROH_20_–_40 Mb_ and ROH_>40 Mb_).

Class (Mb)	Eastern cape (*n* = 20)	Free state (*n* = 24)	Gauteng (*n* = 28)	KwaZulu-natal (*n* = 25)	Limpopo (*n* = 55)	North west (*n* = 55)
0–5 Mb	130	311	390	151	322	645
5–10 Mb	34	146	193	46	100	271
10–20 Mb	8	77	68	34	47	163
20–40 Mb	7	34	19	27	25	72
>40 Mb	1	6	12	6	8	30
Total	180	574	682	264	502	1181

Chromosomal distribution of inbreeding showed higher inbreeding levels in chromosome 15, in Gauteng chromosome 19, in KwaZulu-Natal in chromosome 19 followed by chromosome 16. In the North West, chromosomes 22 and 19 had the highest inbreeding coefficients compared to other chromosomes.

### Distribution of runs of homozygosity

**FIGURE 4:**
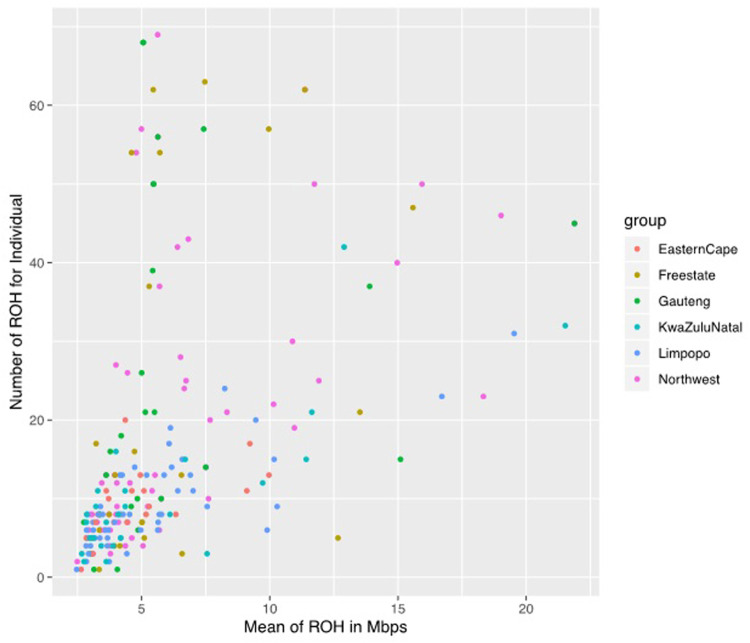
The genomic length with mean runs of homozygosity (ROH) per individual and the number of ROH for individuals.

A total of 3383 ROH were identified across populations, although the frequency and length of ROH differed per population. Among the 207 animals, only 1 animal in the Eastern Cape population was lacking ROH, whilst 206 (99.52%) had at least one ROH longer than 1 Mb. The mean number of ROH per population was 24.36, 23.92, 21.47, 9.13, and 9 in Gauteng, Free State, North West, KwaZulu-Natal, Limpopo, and Eastern Cape, respectively (Figure 4).

The mean ROH length was 7.76 Mb across the population. The longest segment (SNP position 39467151–137516937) was observed in the Gauteng and North West populations and was 98.05 Mb in length (1992 SNPs) found on chromosome 1. In KwaZulu-Natal, Free State and Eastern Cape, the longest segments were found on chromosome 9 (83.54 Mb; 1691 SNPs), chromosome 7 (73.33 Mb; 1449 SNPs) and chromosome 7 (51.81 Mb; 1034 SNPs), respectively. ROH shorter than 5 Mb predominated (*n* = 1949) across all populations (Table 1), accounting for 57.61% of all detected segments and differed per population. These short segments accounted for 72.22% of the Eastern Cape population, followed by Limpopo (*n* = 322; 64%), KwaZulu-Natal (*n* = 151; 57.20%), Gauteng (*n* = 390; 57.18%), North West (*n* = 645; 54.61%), and Free State (*n* = 311; 54.18%).

**FIGURE 5:**
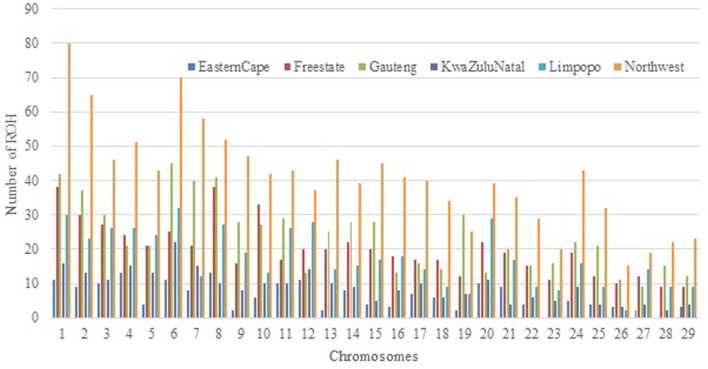
Number of runs of homozygosity (ROH) per chromosome identified across all populations.

The relationship between the mean number of ROH and the length of the genome covered by ROH per individual varies considerably among animals and populations. The number of ROH per chromosome displayed a specific pattern with the larger numbers found for chromosome 1, 2, and 3, a number that tended to decrease with chromosome length, and the smallest number on chromosome (Keller et al., 2011) with 44 segments. Chromosome 1 had the highest number of ROH and the Eastern Cape did not have ROH on chromosomes 23 and 28 (Figure 5).

**FIGURE 6:**
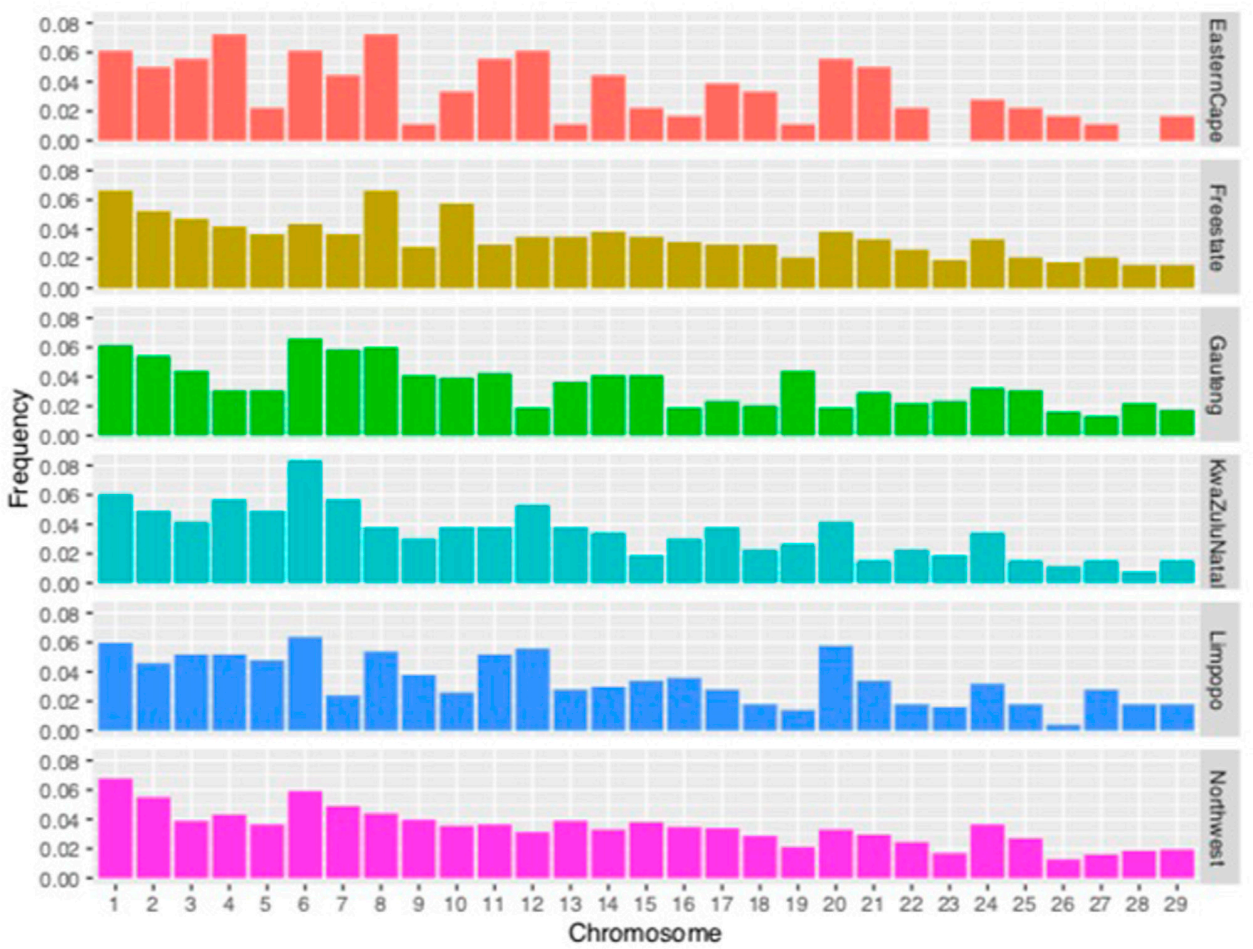
Frequency of runs of homozygosity (ROH) per chromosome per population.

The proportion of chromosomes covered by ROH is illustrated in Figure 6. Overall, the highest coverage by ROH was observed on chromosomes 1, 2, 6, and 8 at 0.37, 0.31, 0.38, and 0.33, respectively. For the Eastern Cape population, chromosomes 4 and 8 had 0.07%, whilst Free State, Limpopo and North West had the highest proportion on chromosome 1 with 0.06%. Gauteng and KwaZulu-Natal had the highest on chromosome 6 with 0.06 and 0.08%, respectively.

**FIGURE 7:**
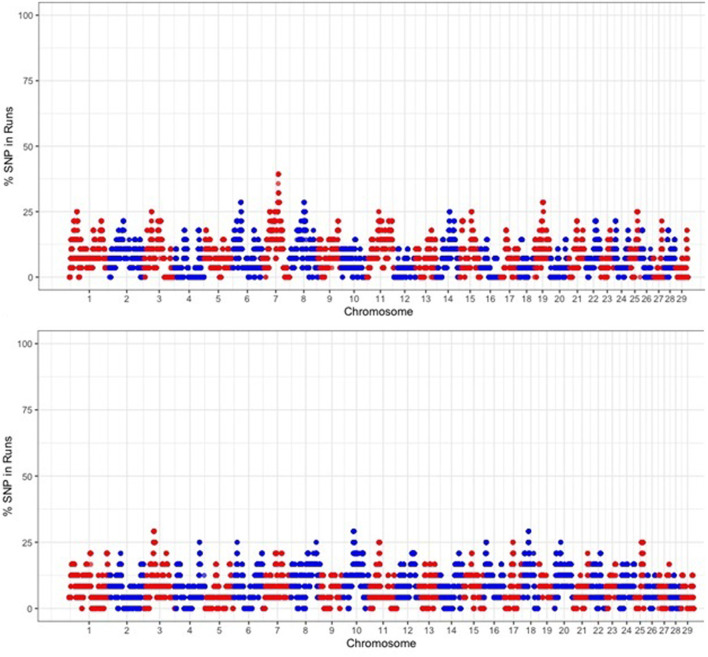
Manhattan plot of occurrences (%) of a SNP in ROH across populations.

To identify the genomic regions that were most commonly associated with ROH, the percentage of SNPs in ROH was assessed by analyzing the frequency of a SNP occurring in those ROH across different individuals (20%), and this was plotted against the position of the SNP along the chromosome (Figure 7). The threshold of 70% and 50% of the individuals did not yield ROH islands across populations, so 20% was used. Several genomic regions were identified that frequently appeared in ROH within individual animals (Table 3). We identified 58 ROH islands at the 20% threshold in the Free State (*n* = 28) and Gauteng (*n* = 29) provinces. No ROH islands were observed in the Eastern Cape, KwaZulu-Natal, North West, and Limpopo at the set thresholds. The ROH hotspot with the highest occurrences (SNPs = 149) in Gauteng was located on chr7 (7.69 Mb). Chromosome position, start and end position of ROH, ROH length and number of SNPs within the genomic regions of extended homozygosity are reported in Table 3 and Additional file 1, Supplementary Table S1. A total of 871 genes are inside the ROH islands, which include important environmental adaptation and hermo-tolerance genes such as *IL10RB, IL23A, FGF9, IGF1, EGR1, MTOR* and *MAPK3*. An additional file 1 shows the KEGG 292 pathways associated with the genes (Supplementary Table S2) that include Fructose and mannose metabolism, Starch and sucrose metabolism, Vitamin B6 metabolism, Protein export and Phototransduction.

**TABLE 3:** Regions of the ROH islands at 20% across Gauteng and Free State populations by length.

Population	Position (chr: SNP 1: SNP 2)	nSNP	Length (Mb)
Gauteng	3:54056151:55070625	22	1.01
Gauteng	15:7897116:9088595	28	1.19
Gauteng	9:76995912:78321101	30	1.33
Gauteng	8:53874425:55257845	34	1.38
Gauteng	19:27892936:29379671	33	1.49
Gauteng	24:7056817:8823189	37	1.77
Gauteng	1:122842771:124683449	34	1.84
Gauteng	15:43908135:45815862	33	1.91
Gauteng	21:30374560:32314473	45	1.94
Gauteng	14:51115518:53088572	38	1.97
Gauteng	25:29189441:31200199	43	2.01
Gauteng	2:53997579:56229103	44	2.23
Gauteng	11:85024580:87384856	47	2.36
Gauteng	8:60260123:62640344	51	2.38
Gauteng	7:29457232:31898006	46	2.44
Gauteng	11:45829634:48502615	52	2.67
Gauteng	1:17079512:19890667	60	2.81
Gauteng	11:95518465:98340959	43	2.82
Gauteng	19:31681378:34691035	57	3.01
Gauteng	1:28400001:31438929	61	3.04
Gauteng	6:15586763:18769652	60	3.18
Gauteng	3:62095554:65301414	62	3.21
Gauteng	8:55933868:59160945	67	3.23
Gauteng	11:50602742:54104265	72	3.50
Gauteng	15:9294238:12900069	70	3.61
Gauteng	22:35705179:39950926	81	4.25
Gauteng	7:45438722:51833004	126	6.39
Gauteng	7:60187788:67883046	149	7.70
Freestate	25:28641340:31924694	69	3.28
Freestate	7:48193142:49278113	19	1.08
Freestate	20:21671108:22811848	22	1.14
Freestate	15:30999298:32153016	30	1.15
Freestate	20:4743026:6211601	32	1.47
Freestate	6:11346351:12878262	32	1.53
Freestate	20:19597454:21199311	37	1.60
Freestate	14:73908424:75554996	30	1.65
Freestate	22:36144242:37987490	36	1.84
Freestate	6:107507640:109477575	40	1.97
Freestate	25:25914911:28121785	48	2.21
Freestate	10:73412812:75622539	47	2.21
Freestate	4:94620783:96868758	55	2.25
Freestate	10:70170521:72471193	48	2.30
Freestate	1:81289909:83642225	50	2.35
Freestate	18:6154744:8563747	54	2.41
Freestate	1:145037345:147663352	55	2.63
Freestate	8:62328607:64991613	62	2.66
Freestate	11:36630830:39372620	53	2.74
Freestate	17:32672670:35522389	54	2.85
Freestate	8:94585706:97590188	52	3.00
Freestate	12:57673212:60742379	61	3.07
Freestate	16:5685888:9117064	69	3.43
Freestate	10:48160488:51636811	64	3.48
Freestate	18:20922080:24775492	76	3.85
Freestate	12:63521630:67564200	87	4.04
Freestate	10:36878056:43333293	124	6.46
Freestate	3:33683337:40785725	140	7.10

### Population structure

**FIGURE 8:**
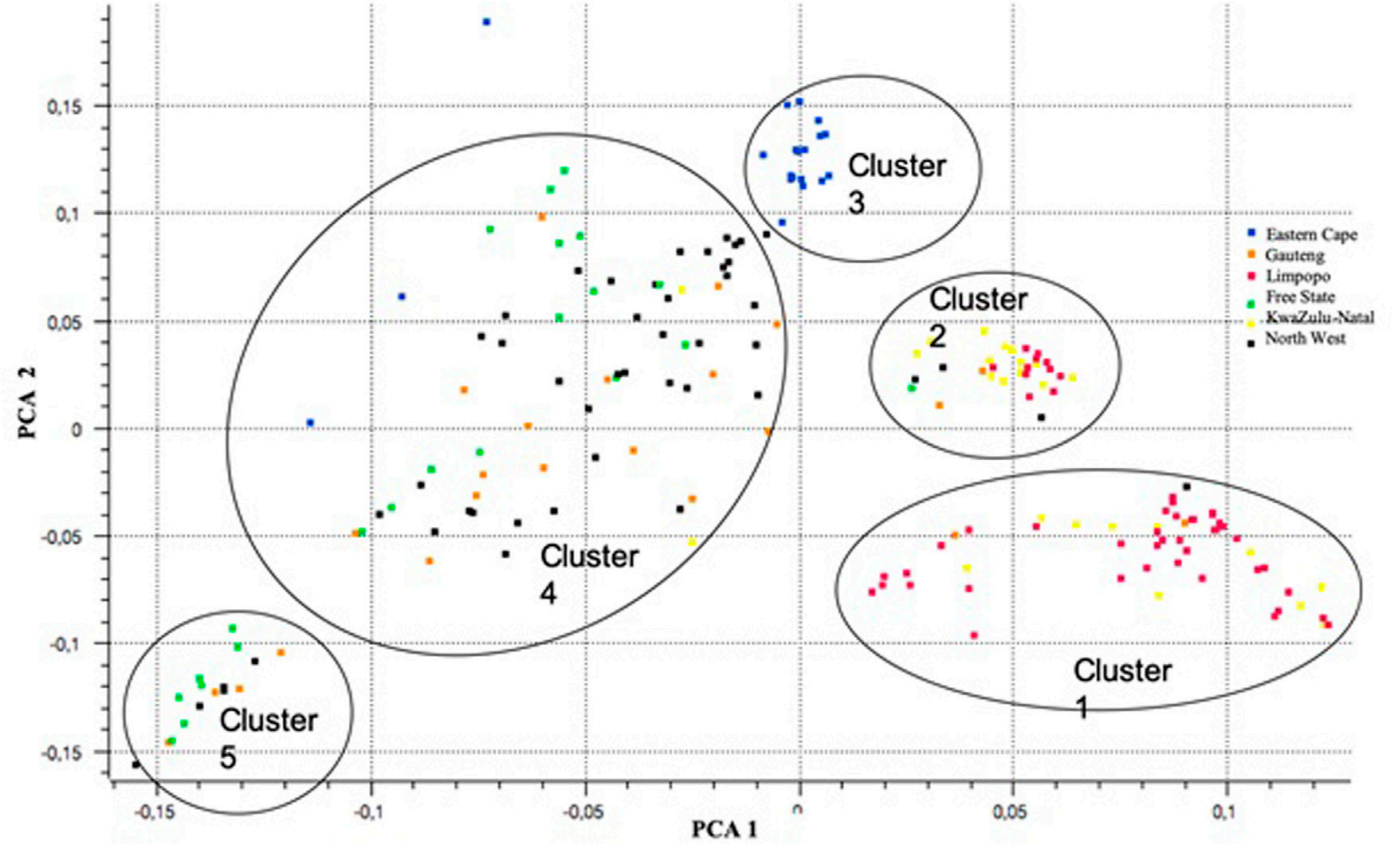
A plot of principal components (PCA) analysis showing differentiations among the clusters of admixtures across six communal indigenous goat populations.

For population structure analysis, further quality control parameters were effected in PLINK v1.90 (Peripolli et al., 2018): to remove linked SNPs using the *--indep-pairwise 50 5 0.5* command and related individuals (IBS > 0.65). The database for population structure included 32886 SNPs and 189 individuals across populations. The plot of the first (PC1) and second eigenvectors (PC2) (Figure 8) shows weak differentiation among the clusters of admixed populations. PC1 shows clusters 1 and 2 consisting of populations from KwaZulu-Natal and Limpopo, respectively. The Eastern Cape population (Cluster 3) revealed a separate cluster (with three outliers). Clusters 4 consists of the majority of the North West and Gauteng, whilst cluster 5, was Free State.

**FIGURE 9:**
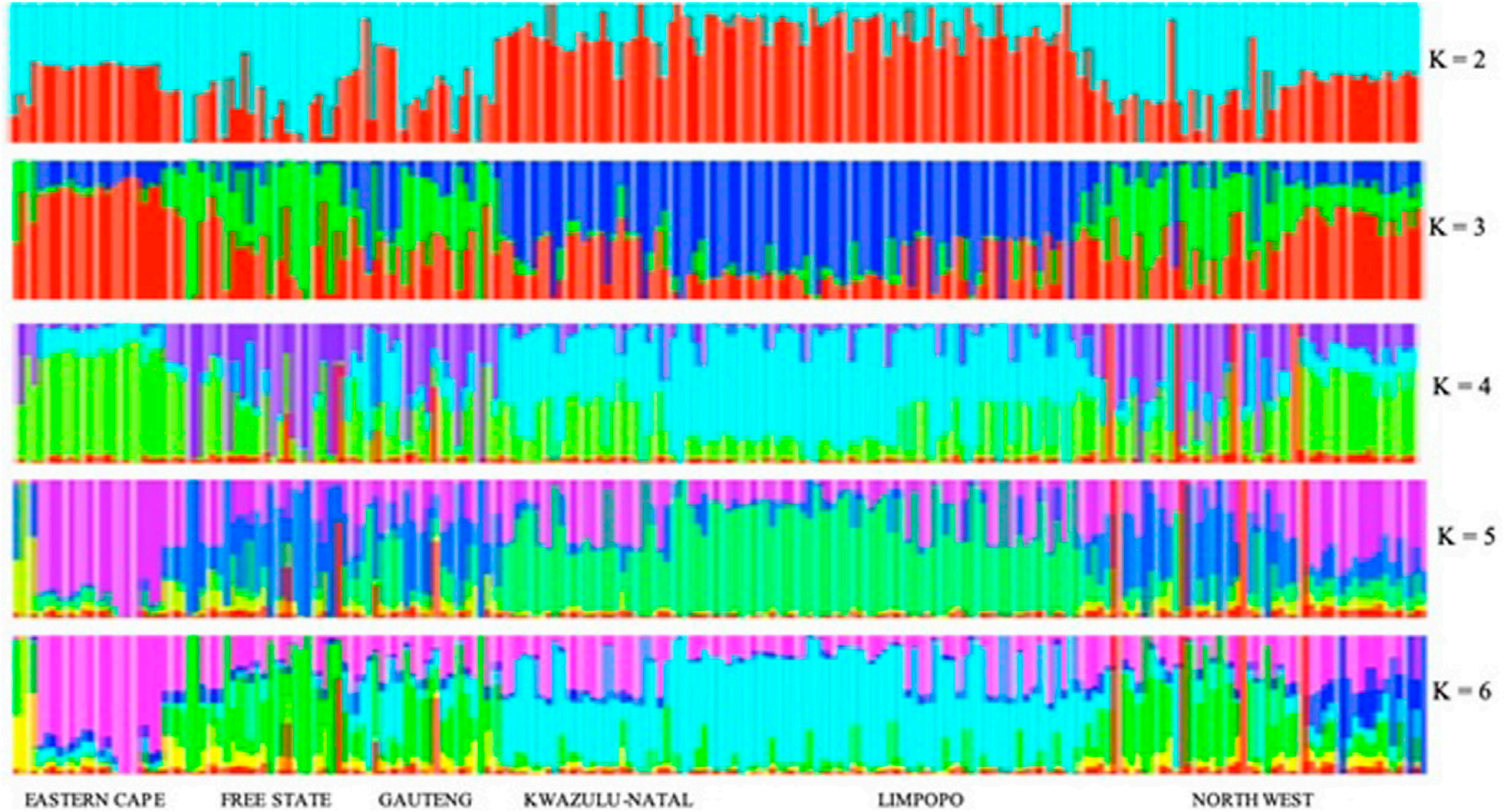
Clusters inferred from ADMIXTURE at K = 2–6.

For the further understanding of the degree of admixture within the populations, the ADMIXTURE 1.3 (Alexander et al., 2009) software was used for K = 2 to 10 hypothetical ancestral populations, only K = 2 to K = 6 is plotted since no further clusters were observed after K = 6 (Figure 9). Assuming two hypothetical ancestral populations, the populations from KwaZulu-Natal and Limpopo are revealed, supporting PC 1. The genomes of KwaZulu-Natal and Limpopo share an origin but have substantial admixture from the Eastern Cape and North West populations. K = 3 as the most likely number of genetically distinct groups within our populations, presenting the lowest cross-validation error (0.4617), reflecting a clear cluster of the Limpopo populations. The Free State, Gauteng, and North West showed similar genetic heterogeneity patterns with a considerable level of admixture. The North West revealed a high level of within-population genetic differentiation as there are individuals closer to the Eastern Cape and another subpopulation closer or clustering with Free State. This is also in agreement with the second PC coordinate analysis in showing genetic heterogeneity within the population. Moreover, with the increment of the value of K (K = 4 to K = 6), Free State and Gauteng show a higher level of genetic heterogeneity than the other populations.

## Discussion

In this study, the genetic diversity and population structure of goat populations in South Africa were revealed. The main links to the development of commercial goat populations are represented by indigenous goat populations and may potentially be relevant as a future source of untapped adaptable genetic material (Webb and Mamabolo, 2004). Therefore, improving our understanding of within-ecotype relationships among the major goat producing provinces in South Africa offers a rare opportunity to enhance efficient use of the breeds and implement conservation programs. This study investigated the indications for population status on inbreeding and runs of homozygosity in the indigenous goat population. Data from a previous study (Mdladla et al., 2018) enabled a broad geographical coverage of South Africa and represented populations from the major goat producing provinces within the country.

Effective population size (*N*_*e*_) is a crucial population genetic parameter because of its relationship to the loss of genetic variation, increases in inbreeding, the accumulation of mutations, and the determination of the accuracy of genomic selection (Goddard, 2009; Berihulay et al., 2019). Gauteng had the smallest estimated *N*_*e*_ among the population, and Limpopo had the highest. It was also observed in other studies that effective population size (*N*_*e*_) showed a reduction to 132 in the Kingdom of Eswatini and the highest in South Africa 12 generations ago (Webb and Mamabolo, 2004). It is recommended that to prevent a reduction of the adaptive value in populations, *N*_*e*_ values between 50 and 100 animals (Meuwissen and Goddard, 2007) and 50 to avoid inbreeding depression. A study by (Colli et al., 2018), reported a large *N*_*e*_ in local goat breeds, such as those from Africa, Spain, and Central-Southern Italy, local goats breeds and a small *Ne* in the Angora, Boer, Nubian, Cashmere, Saanen, and Alpine populations.

The rapid increase pattern in *Ne* may also include bottlenecks associated with domestication, selection, and breed formation, as well as the endangerment of the breed (Shin et al., 2013). A study by (Mdladla, 2016) based on SNP data and using the same method, reported large *N*_*e*_ for all investigated breeds (ranging from 140 to 348). Furthermore, a study by (Mdladla et al., 2016a) revealed that the ecotype goat had a slightly higher effective population size than the Tankwa and commercial goat populations across generations. From a conservation standpoint, the indigenous goat population should be top priority in the population studied due to their diminishing effective population size and increased inbreeding coefficients.

Runs of homozygosity (ROH) can disclose the genetic relationships among individuals, estimating with high accuracy the autozygosity at the individual and population levels and can elucidate about selection pressure events (Purfield et al., 2012). If long ROH accumulates in the genomes of some individuals, they could seriously impact the overall biological fitness (Manunza et al., 2016). Therefore, it was an important objective to investigate and understand the level of homozygosity among the populations. In this study, only 1 animal was lacking ROH, whilst 206 (99.52%) had at least one ROH longer than 1 Mb. The genomic inbreeding coefficients (*F*_*ROH*_) values found in the study for the Ethiopian goats were *F*_*ROH* > 1 Mb_ values (Nandolo et al., 2019). Similar results were found by (Nandolo et al., 2019) with more African goats (Cameroon, Ethiopia, Kenya, Madagascar, Malawi, Mali, Mozambique, Nigeria, Tanzania, Uganda, and Zimbabwe) using a clustering algorithm. In this study, differences in terms of total number and length of ROH were short (>5 Mb) were more abundant (57.61%) (Onzima et al., 2018) reported results that showed lower than the *F*_*ROH* > 2 Mb_ for Kenya, Uganda, and Mozambique goat breeds when the Goat 50K BeadChip was used. On the other hand, the Eastern Cape population showed very low amounts of ROH. This has been suggested to be consistent with recent admixture in the individuals of Chinese cattle (Xu et al., 2019). Long segments were abundant in the North West population (Islam et al., 2019). A recent study revealed a high mean ROH in the long length category (>30 Mb), and their study suggested that inbreeding is more recent and is indicative of demographic decline. Considering the extensive management systems of goats in the region, these results might be likely even if some researchers have argued that such extensive systems may lead to inbreeding, especially where goats are on an extensive system or are shepherded with other flocks for some part of the year (Tefera et al., 2004; Rumosa Gwaze et al., 2009b). The lower inbreeding levels in African goats could be due to the openness of the breeding systems in most of Africa that leads to the loose definition of livestock breeds in the region (Manunza et al., 2016).

One of the main advantages of genomic coefficients is the availability of chromosomal inbreeding coefficients (McQuillan et al., 2008; Mastrangelo et al., 2016). ROHs, representing the level of genomic autozygosity, are continuous homozygous segments at the individual and population levels that can be used as a measurement of inbreeding; more in-depth ROHs are the result of demography, natural and artificial selection, and inbreeding (Shi and Manley, 2007). In this study, we do not discuss in detail all the genomic regions associated with ROH but focus on some selected regions that show associations with several specific traits related to livestock breeding. We identified five genes reported to be associated with the important traits of goats (Figure 6) identified by the selection signature. Overall, the highest coverage by ROH was observed on chromosomes 1, 2,and 6, respectively. Gene INHA, located on chromosome 2, was reported as a candidate gene for litter size in goats (Hou et al., 2012). Significant QTL for milk production traits such as milk yield and milk protein have been reported on chromosome 2 in sheep (Garcia-Gámez et al., 2013). Gene INHA, located on chromosome 2, was reported as a candidate gene for litter size in goats. The *PPP1R36* and Heat Shock Protein A2 (*HSPA2*) (*CHI10*, 26.402–26.719 Mb) identified in these communal indigenous goats are involved in heat stress response and, in other studies, *HSPA2*, *DNAJC24*, and *DNAJC*13 are associated with the heat shock family of genes (Shi and Manley, 2007). The presence of multiple genes associated with heat stress would seem to suggest that the trait is under intense selection pressure in tropically adapted breeds (Onzima et al., 2018).

In accordance with (Nothnagel et al., 2010), these regions in humans, when they are present in more than 50% of the individuals of a population, can indicate a strong selection occurrence. The occurrence of ROH hotspots in genomic regions that harbor candidate genes may be involved in selection pressure in response to production and environmental conditions. This study identified 58 ROH hotspots in Gauteng and Free State populations and revealed 871 genes and 292 KEGG pathways. This threshold did not yield any results, and only about 20% of ROH islands were detected in the communal indigenous goat population. The 20% threshold has been used in indigenous Chinese pigs, Jinhua (Xu et al., 2019). ROH islands can be defined as genomic regions with reduced genetic diversity and, consequently, high homozygosity around the selected locus that might harbor targets of positive selection and are under strong selective pressure (Pemberton et al., 2012; Peripolli et al., 2018). The minimum expected length of homozygous DNA segments is based on the time frame of approximately 25 generations, over which goats are believed to have been characterized into separate breeds (Onzima et al., 2018).

This study explored the population genetic structure of the indigenous goat population in the context of all South African goat populations. In accordance with our earlier studies (Lashmar et al., 2016; Mdladla et al., 2017), the principal component analysis (PCA), and the ADMIXTURE analyses based on the SNP array and sequence data sets, capitulated the major genetic division among the South African goat populations from two large geographic regions: Eastern Cape and Limpopo. The KwaZulu-Natal and Limpopo populations’ genomes shared an origin yet with significant admixture from the Eastern Cape and North West populations. Some signals of admixture and underlying genetic relationships among the populations were generated by analysis of population admixture (Hou et al., 2012). A migration route of ancient goat from the northern part of South Africa to the eastern areas of the KZN, during their migration periods of the Bantu nation, is supported by this study as observed.

## Conclusion

The results of this study indicated a greater negative impact of inbreeding in recent times, which is important for planning conservation strategies. It was revealed that the occurrence of high *N*_*e*_ and autozygosity varied largely across ecotypes in communal indigenous goat populations at recent and ancient events when a genome-wide SNP marker was used. The use of Illumina goat SNP50K BeadChip shows that there was a migration route of communal indigenous goat populations from the northern part (Limpopo province) of South Africa to the eastern areas of KwaZulu-Natal, which confirmed their historical relatedness and coincides with the migration periods of the Bantu nation. The communal-traditional indigenous goat farming system and adaptation to different climatic conditions had an influence on the results in this study. The study deepened the understanding of the conservation status and selection mechanisms of goats in a communal indigenous goat production setting. For effective conservation programs and utilization of South African communal indigenous goat populations, effort should be made to establish a conservation program for the unique genetic resources of indigenous goat populations.

## Data Availability

The datasets presented in this study can be found in online repositories. The name of the repository and link to the data can be found at: Dryad; https://doi.org/10.5061/dryad.931zcrjnh.

## References

[B1] Ai H. Huang L. Ren J. (2013). Genetic diversity, linkage disequilibrium and selection signatures in Chinese and Western pigs revealed by genome-wide SNP markers. PLoS One 8 (2), e56001. 23409110 10.1371/journal.pone.0056001 PMC3567019

[B2] Alexander D. H. Novembre J. Lange K. (2009). Fast model-based estimation of ancestry in unrelated individuals. Genome. Res. 19, 1655–1664. 19648217 10.1101/gr.094052.109 PMC2752134

[B3] Alvarenga A. B. Rovadoscki G. A. Petrini J. Coutinho L. L. Morota G. Spangler M. L. (2018). Linkage disequilibrium in Brazilian Santa Inês breed, *Ovis aries* . Sci. Rep. 8, 8851. 10.1038/s41598-018-27259-7 29892085 PMC5995818

[B4] Barbato M. Orozco-Terwengel P. Tapio M. Bruford M. W. (2015). SNeP: A tool to estimate trends in recent effective population size trajectories using genome-wide SNP data. Front. Genet. 6, 109. 10.3389/fgene.2015.00109 25852748 PMC4367434

[B5] Berihulay H. Islam R. Jiang L. Ma Y. (2019). Genome-wide linkage disequilibrium and the extent of effective population sizes in six Chinese goat populations using a 50K single nucleotide polymorphism panel. Animals 9, 350. 10.3390/ani9060350 PMC6617254 31200540

[B6] Biscarini F. Cozzi P. Gaspa G. Marras G. (2018). bioinformatics. Available at: https://github.com/bioinformatics-ptp/detectRUNS/tree/master/detectRUNS .

[B7] Bosse M. Megens H.-J. Madsen O. Paudel Y. Frantz L. A. F. Schook L. B. (2012). Regions of homozygosity in the porcine genome: Consequence of demography and the recombination landscape. PLOS Genet. 8, e1003100. 10.1371/journal.pgen.1003100 23209444 PMC3510040

[B8] Broman K. W. Weber J. L. (1999). Long homozygous chromosomal segments in reference families from the centre d'Étude du Polymorphisme humain. Am. J. Hum. Genet. 65, 1493–1500. 10.1086/302661 10577902 PMC1288359

[B9] Buchmann R. Hazelhurst S. (2014). Genesis manual. Johannesburg: University of the Witwatersrand.

[B10] Colli L. Milanesi M. Milanesi M. Talenti A. Bertolini F. Chen M. (2018). Genome-wide SNP profiling of worldwide goat populations reveals strong partitioning of diversity and highlights post-domestication migration routes. Genet. Sel. Evol. 50, 58. 10.1186/s12711-018-0422-x 30449284 PMC6240949

[B11] Curik I. Ferenčaković M. Sölkner J. (2014). Inbreeding and runs of homozygosity: A possible solution to an old problem. Livest. Sci. 166, 26–34. 10.1016/j.livsci.2014.05.034

[B12] Deng T. Liang A. Liu J. Hua G. Ye T. Liu S. (2019). Genome-wide SNP data revealed the extent of linkage disequilibrium, persistence of phase and effective population size in purebred and crossbred buffalo populations. Front. Genet. 9, 688. 10.3389/fgene.2018.00688 30671082 PMC6332145

[B13] Dong Y. Xie M. Jiang Y. Xiao N. Du X. Zhang W. (2013). Sequencing and automated whole-genome optical mapping of the genome of a domestic goat (*Capra hircus*). Nat. Biotechnol. 31, 135–141. 10.1038/nbt.2478 23263233

[B14] Dube K. (2015). Characterization of goat production systems in selected coastal areas of the Eastern Cape Province, South Africa. PhD Thesis.

[B15] Ferenčaković M. Hamzic E. Gredler B. Curik I. Sölkner J. (2011). Runs of homozygosity reveal genome-wide autozygosity in the Austrian fleckvieh cattle. Agric. Conspec. Sci. 76, 325–328.

[B16] Ferenčaković M. Hamzić E. Gredler B. Solberg T. R. Klemetsdal G. Curik I. (2013). Estimates of autozygosity derived from runs of homozygosity: Empirical evidence from selected cattle populations. J. Anim. Breed. Genet. 130, 286–293. 10.1111/jbg.12012 23855630

[B17] Flury C. Tapio M. Sonstegard T. Drögemüller C. Leeb T. Simianer H. (2010). Effective population size of an indigenous Swiss cattle breed estimated from linkage disequilibrium. J. Anim. Breed. Genet. 127, 339–347. 10.1111/j.1439-0388.2010.00862.x 20831557

[B18] Food and Agriculture Organization of the United Nations (2015b). Draft second report on the state of the world’s animal genetic resources for food and agriculture (Part3), commission on genetic resources for food and agriculture, (CGRFA-15/15/Inf.17.2). Rome. From: Available at: http://www.fao.org/3/a-mm310e.pdf (Accessed August 24, 2018).

[B19] Garcia-Gámez E. Gutiérrez-Gil B. Suarez-Vega A. de la Fuente L. F. Arranz J. J. (2013). Identification of quantitative trait loci underlying milk traits in Spanish dairy sheep using linkage plus combined linkage disequilibrium and linkage analysis approaches. J. Dairy Sci. 96, 6059–6069. 23810588 10.3168/jds.2013-6824

[B20] Gibson J. Morton N. E. Collins A. (2006). Extended tracts of homozygosity in outbred human populations. Hum. Mol.Genet. 15, 789–795. 10.1093/hmg/ddi493 16436455

[B21] Goddard M. (2009). Genomic selection: Prediction of accuracy and maximisation of long term response. Genetica 136, 245–257. 10.1007/s10709-008-9308-0 18704696

[B22] Hou J. An X. Li G. Wang Y. Song Y. Cao B. (2012). Exploring polymorphisms and their effects on reproductive traits of the INHA and INHβA genes in three goat breeds. Anim. Sci. J. 83, 273–278. 10.1111/j.1740-0929.2011.00968.x 22515685

[B23] Howrigan D. P. Simonson M. A. Keller M. C. (2011). Detecting autozygosity through runs of homozygosity: A comparison of three autozygosity detection algorithms. BMC Genomics 12, 460. 10.1186/1471-2164-12-460 21943305 PMC3188534

[B24] Islam R. Li Y. Liu X. Berihulay H. Abied A. Gebreselassie G. (2019). Genome-wide runs of homozygosity, effective population size, and detection of positive selection signatures in six Chinese goat breeds. Genes. 10, 938. 10.3390/genes10110938 PMC6895971 31744198

[B25] Keller M. C. Visscher P. M. Goddard M. E. (2011). Quantification of inbreeding due to distant ancestors and its detection using dense single nucleotide polymorphism data. Genetics 189, 237–249. 10.1534/genetics.111.130922 21705750 PMC3176119

[B26] Kotzé A. Grobler J. Van Marle-Köster E. Jonker T. Dalton D. (2014). The Tankwa karoo national park feral goat population: A unique genetic resource. SA J. An. Sci. 44 (1), 43–48. 10.4314/sajas.v44i1.6

[B27] Kotze A. Swart H. Grobler J. P. Nemaangani A. (2004). A genetic profile of the Kalahari Red goat breed from Southern Africa. S A. J. Anim. Sci. 34, 10–12.

[B28] Lashmar S. F. Visser C. Marle-Köster E. v. (2016). SNP-based genetic diversity of South African commercial dairy and fiber goat breeds. Small Ruminant Res. 136, 65–71. 10.1016/j.smallrumres.2016.01.006

[B29] Liu S. He S. Chen L. Li W. Di J. Liu M. (2017). Estimates of linkage disequilibrium and effective population sizes in Chinese Merino (Xinjiang type) sheep by genome-wide SNPs. Genes. Genom 39, 733–745. 10.1007/s13258-017-0539-2 PMC5486679 28706593

[B30] Manunza A. Noce A. Serradilla J. M. Goyache F. Martínez A. Capote J. (2016). A genome-wide perspective about the diversity and demographic history of seven Spanish goat breeds. Genet. Sel. Evol. 48, 52. 10.1186/s12711-016-0229-6 27455838 PMC4960707

[B31] Marras G. Gaspa G. Sorbolini S. Dimauro C. Ajmone-Marsan P. Valentini A. (2014). Analysis of runs of homozygosity and their relationship with inbreeding in five cattle breeds farmed in Italy. Anim. Genet. 46, 110–121. 10.1111/age.12259 25530322

[B32] Martin P. M. Palhière I. Ricard A. Tosser-Klopp G. Rupp R. (2016). Genome wide association study identifies new loci associated with undesired coat color phenotypes in saanen goats. PLoS ONE 11, e0152426. 10.1371/journal.pone.0152426 27030980 PMC4816504

[B33] Mastrangelo S. Tolone M. Di Gerlando R. Fontanesi L. Sardina M. T. Portolano B. (2016). Genomic inbreeding estimation in small populations: Evaluation of runs of homozygosity in three local dairy cattle breeds. Animal 10, 746–754. 10.1017/s1751731115002943 27076405

[B34] Mastrangelo S. Tolone M. Sardina M. T. Sottile G. Sutera A. M. Di Gerlando R. (2017). Genome-wide scan for runs of homozygosity identifies potential candidate genes associated with local adaptation in Valle del Belice sheep. Genet. Sel. Evol. 49 (1), 84. 10.1186/s12711-017-0360-z.hal-01635193 29137622 PMC5684758

[B35] McQuillan R. Leutenegger A. L. Abdel-Rahman R. Franklin C. S. Pericic M. Barac-Lauc L. (2008). Runs of homozygosity in European populations. Am. J. Hum. Genet. 83 (3), 359–372. 10.1016/j.ajhg.2008.08.007 18760389 PMC2556426

[B36] Mdladla K. Dzomba E. F. Huson H. J. Muchadeyi F. C. (2016a). Population genomic structure and linkage disequilibrium analysis of South African goat breeds using genome-wide SNP data. Anim. Genet. 47, 471–482. 10.1111/age.12442 27306145

[B37] Mdladla K. Dzomba E. F. Muchadeyi F. C. (2017). Characterization of the village goat production systems in the rural communities of the eastern Cape, KwaZulu-natal, Limpopo and North West provinces of South Africa. Trop. Anim. Health Prod. 49, 515–527. 10.1007/s11250-017-1223-x 28150112

[B38] Mdladla K. Dzomba E. F. Muchadeyi F. C. (2018). Landscape genomics and pathway analysis to understand genetic adaptation of South African indigenous goat populations. Heredity 120 (4), 369–378. 10.1038/s41437-017-0044-z 29422506 PMC5842216

[B39] Mdladla K. (2016). Landscape genomic approach to investigate genetic adaptation in South African indigenous goat populations. PhD thesis. 10.1038/s41437-017-0044-z PMC5842216 29422506

[B40] Meuwissen T. H. E. Goddard M. E. (2007). Multipoint identity-by-descent prediction using dense markers to map quantitative trait loci and estimate effective population size. Genetics 176, 2551–2560. 10.1534/genetics.107.070953 17565953 PMC1950654

[B41] Monau P. Raphaka K. Zvinorova-Chimboza P. Gondwe T. (2020). Sustainable utilization of indigenous goats in southern Africa. Diversity 12, 20. 10.3390/d12010020

[B42] Morrison J. W. (2007). A guide to the identification of the natural indigenous goats of Southern Africa. From: http://www.landbou.com/wp.../03/f2297405-a93f-4399-bdb7-6f3de538d75d.pdf (Accessed April 20, 2018).

[B43] Mthi S. Skenjana A. Fayemi P. O. (2017). Characteristics of small-scale sheep production systems in some communal areas of the Eastern Cape Province, South Africa. Int. J. Livest. Prod. 8, 199–206.

[B44] Nandolo W. Mészáros G. Banda L. J. Gondwe T. N. Lamuno D. Mulindwa H. A. (2019). Timing and extent of inbreeding in african goats. Front. Genet. 10, 537. 10.3389/fgene.2019.00537 31214253 PMC6558083

[B45] Nicoloso L. Bomba L. Bomba L. Colli L. Negrini R. Milanesi M. (2015). Genetic diversity of Italian goat breeds assessed with a medium-density SNP chip. Genet. Sel. Evol. 47, 62. 10.1186/s12711-015-0140-6 26239391 PMC4523021

[B46] Nothnagel M. Lu T. T. Kayser M. Krawczak M. (2010). Genomic and geographic distribution of SNP-defined runs of homozygosity in Europeans. Hum. Mol.Genet. 19, 2927–2935. 10.1093/hmg/ddq198 20462934

[B47] Nyamushamba G. B. Mapiye C. Tada O. Halimani T. E. Muchenje V. (2017). Conservation of indigenous cattle genetic resources in southern africa's smallholder areas: Turning threats into opportunities - a review. Asian-Australas J. Anim. Sci. 30, 603–621. 10.5713/ajas.16.0024 27004814 PMC5411820

[B48] Onzima R. B. Upadhyay M. R. Doekes H. P. Brito L. F. Bosse M. Kanis E. (2018). Genome-wide characterization of selection signatures and runs of homozygosity in Ugandan goat breeds. Front. Genet. 9, 318. 10.3389/fgene.2018.00318 30154830 PMC6102322

[B49] Ouborg N. J. Pertoldi C. Loeschcke V. Bijlsma R. Hedrick P. W. (2010). Conservation genetics in transition to conservation genomics. Trends Genet. 26, 177–187. 10.1016/j.tig.2010.01.001 20227782

[B50] Pemberton T. J. Absher D. Feldman M. W. Myers R. M. Rosenberg N. A. Li J. Z. (2012). Genomic patterns of homozygosity in worldwide human populations. Am. J. Hum. Genet. 91, 275–292. 10.1016/j.ajhg.2012.06.014 22883143 PMC3415543

[B51] Peripolli E. Stafuzza N. B. Munari D. P. Lima A. L. F. Irgang R. Machado M. A. (2018). Assessment of runs of homozygosity islands and estimates of genomic inbreeding in Gyr (*Bos indicus*) dairy cattle. BMC Genomics 19, 34. 10.1186/s12864-017-4365-3 29316879 PMC5759835

[B52] Purcell S. Neale B. Todd-Brown K. Thomas L. Ferreira M. A. R. Bender D. (2007). Plink: A tool set for whole-genome association and population-based linkage analyses. Am. J. Hum. Genet. 81, 559–575. 10.1086/519795 17701901 PMC1950838

[B53] Purfield D. C. Berry D. P. McParland S. Bradley D. G. (2012). Runs of homozygosity and population history in cattle. BMC Genet. 13, 70. 10.1186/1471-2156-13-70 22888858 PMC3502433

[B54] Purfield D. C. McParland S. Wall E. Berry D. P. (2017). The distribution of runs of homozygosity and selection signatures in six commercial meat sheep breeds. PLoS One 12, e0176780. 10.1371/journal.pone.0176780 28463982 PMC5413029

[B55] Rumosa Gwaze F. Chimonyo M. Dzama K. (2009b). Communal goat production in southern Africa: A review. Trop. Anim. Health Prod. 41, 1157–1168. 10.1007/s11250-008-9296-1 19083117

[B56] Shi Y. Manley J. L. (2007). A complex signaling pathway regulates SRp38 phosphorylation and pre-mRNA splicing in response to heat shock. Mol. Cell. 28, 79–90. 10.1016/j.molcel.2007.08.028 17936706

[B57] Shin D.-H. Cho K.-H. Park K.-D. Lee H.-J. Kim H. (2013). Accurate estimation of effective population size in the Korean dairy cattle based on linkage disequilibrium corrected by genomic relationship matrix. Asian Australas. J. Anim. Sci. 26, 1672–1679. 10.5713/ajas.2013.13320 25049757 PMC4092893

[B58] Silió L. Rodríguez M. C. Fernández A. Barragán C. Benítez R. Óvilo C. (2013). Measuring inbreeding and inbreeding depression on pig growth from pedigree or SNP-derived metrics. J. Anim. Breed. Genet. 130, 349–360. 24074172 10.1111/jbg.12031

[B59] Tefera A. N. Mekala D. G. Mnisi P. E. Mukisira M. Clerkson M. Murungweni C. (2004). Goat production and livelihood systems in the Sekhukhune district of the Limpopo Province, South Africa: Opportunities for commercializing goats and their by-products. Work. Doc. Ser. 118. Available at; http://search.ebscohost.com/login.aspx?direct=true&db=lah&AN= 20043182451&site=ehost-live (accessed October 25, 2019).

[B60] Tosser-Klopp G. Bardou P. Bouchez O. Cabau C. Crooijmans R. Dong Y. (2014). Design and characterization of a 52K SNP chip for goats. PloS One 9, e86227. 10.1371/journal.pone.0086227 24465974 PMC3899236

[B61] Visser C. Hefer C. A. van Marle-Köster E. V. Kotze A. (2004). Genetic variation of three commercial and three indigenous goat populations in South Africa. S A. J. Anim. Sci. 34, 24–27.

[B62] Visser C. Lashmar S. F. Van Marle-Köster E. Poli M. A. Allain D. (2016). Genetic diversity and population structure in South African, French and argentinian Angora goats from genome-wide SNP data. PLoS One 11–e0154353. 10.1371/journal.pone.0154353 PMC4865245 27171175

[B63] Webb E. C. Mamabolo M. J. (2004). Production and reproduction characteristics of South African indigenous goats in communal farming systems. Sa. J. Anim. Sci. 34 (1), 236–239.

[B64] Xu L. Zhao G. Yang L. Zhu B. Chen Y. Zhang L. (2019). Genomic patterns of homozygosity in Chinese local cattle. Sci. Rep. 9 (1), 16977. 10.1038/s41598-019-53274-3 31740716 PMC6861314

[B65] Zhang Q. Guldbrandtsen B. Bosse M. Lund M. S. Sahana G. (2015). Runs of homozygosity and distribution of functional variants in the cattle genome. BMC Genomics 16, 542. 10.1186/s12864-015-1715-x 26198692 PMC4508970

[B66] Zhao F. Wang G. Zeng T. Wei C. Zhang L. Wang H. (2014). Estimations of genomic linkage disequilibrium and effective population sizes in three sheep populations. Livest. Sci. 170, 22–29. 10.1016/j.livsci.2014.10.015

[B67] Zvinorova P. I. (2017). A genome-wide association study on mechanisms underlying genetic resistance to gastrointestinal parasites in goats. Ph.D. thesis. Stellenbosch.

