# Supplementary material for: Conservation status and historical relatedness of South African communal indigenous goat populations using a genome-wide single-nucleotide polymorphism marker
Source: Front Genet. 2022 Aug 9;13:909472. doi: 10.3389/fgene.2022.909472 (PMC9395594; doi:10.3389/fgene.2022.909472)
Supplement: Supplementary file 1 [file DataSheet1.docx]

**FILE 1**

**TABLE S 1**: GENES AND GENES DESCRIPTIONS BASED ON REGIONS WITH HIGH FREQUENCY OF ROH

**Gene name Gene description**

5S_rRNA 5S ribosomal RNA

7SK 7SK RNA

ABCA7 ATP binding cassette subfamily A member 7

ABCC5 ATP binding cassette subfamily C member 5

ABCF3 ATP binding cassette subfamily F member 3

ABHD17A abhydrolase domain containing 17A

ABLIM3 actin binding LIM protein family member 3

ACCS 1-aminocyclopropane-1-carboxylate synthase homolog (inactive)

ACCSL 1-aminocyclopropane-1-carboxylate synthase homolog (inactive) like

ACTBL2 actin beta like 2

ADAM10 ADAM metallopeptidase domain 10

ADAMTS9 ADAM metallopeptidase with thrombospondin type 1 motif 9

ADORA2B adenosine A2b receptor

AFAP1L1 actin filament associated protein 1 like 1

AFF4 AF4/FMR2 family member 4

AGMO alkylglycerol monooxygenase

AGR2 anterior gradient 2, protein disulphide isomerase family member

AGR3 anterior gradient 3, protein disulphide isomerase family member

AHSP alpha hemoglobin stabilizing protein

AK1 adenylate kinase 1

AK4 adenylate kinase 4

AK5 adenylate kinase 5

AKAP10 A-kinase anchoring protein 10

AKTIP AKT interacting protein

ALDH1A2 aldehyde dehydrogenase 1 family member A2

ALDH3A1 aldehyde dehydrogenase 3 family member A1

ALG11 ALG11 alpha-1,2-mannosyltransferase

ALG2 ALG2 alpha-1,3/1,6-mannosyltransferase

ALG3 ALG3 alpha-1,3- mannosyltransferase

ALG6 ALG6 alpha-1,3-glucosyltransferase

ALKBH3 alkB homolog 3, alpha-ketoglutaratedependent dioxygenase

ALKBH5 alkB homolog 5, RNA demethylase

ALX4 ALX homeobox 4

ANAPC15 anaphase promoting complex subunit 15

ANGPTL2 angiopoietin like 2

ANGPTL3 angiopoietin like 3

ANK2 ankyrin 2

ANKMY2 ankyrin repeat and MYND domain containing 2

ANKRD34B ankyrin repeat domain 34B

ANKS6 ankyrin repeat and sterile alpha motif domain containing 6

ANXA6 annexin A6

AP2M1 adaptor related protein complex 2 subunit mu 1

APC2 APC regulator of WNT signaling pathway 2

API5 apoptosis inhibitor 5

AQP9 aquaporin 9

**TABLE S 1**: GENES AND GENES DESCRIPTIONS BASED ON REGIONS WITH HIGH FREQUENCY OF ROH (Continued)

**Gene name Gene description**

ARHGEF37 Rho guanine nucleotide exchange factor 37

ARID3A AT-rich interaction domain 3A

ARL11 ADP ribosylation factor like GTPase 11

ARMC5 armadillo repeat containing 5

ARSI arylsulfatase family member I

ARSJ arylsulfatase family member J

ART1 ADP-ribosyltransferase 1

ASL argininosuccinate lyase

ASPHD1 aspartate beta-hydroxylase domain containing 1

ATG4C autophagy related 4C cysteine peptidase

ATOH1 atonal bHLH transcription factor 1

ATP5F1D ATP synthase F1 subunit delta

ATP6V0E1 ATPase H+ transporting V0 subunit e1

ATP6V1C2 ATPase H+ transporting V1 subunit C2

ATP7B ATPase copper transporting beta

ATP8B3 ATPase phospholipid transporting 8B3

ATP8B4 ATPase phospholipid transporting 8B4 (putative)

ATPAF2 ATP synthase mitochondrial F1 complex assembly factor 2

AUTS2 activator of transcription and developmental regulator AUTS2

AZU1 azurocidin 1

B3GNT5 UDP-GlcNAc:betaGal beta-1,3-N-acetylglucosaminyltransferase 5

B9D1 B9 domain containing 1

BCKDK branched chain keto acid dehydrogenase kinase

BCL10 BCL10 immune signaling adaptor

BCL7C BAF chromatin remodeling complex subunit BCL7C

BHLHE22 basic helix-loop-helix family member e22

BLOC1S6 biogenesis of lysosomal organelles complex 1 subunit 6

BNIP1 BCL2 interacting protein 1

BRCA2 BRCA2 DNA repair associated

BRD8 bromodomain containing 8

BTBD2 BTB domain containing 2

BTG3 BTG anti-proliferation factor 3

BZW2 basic leucine zipper and W2 domains 2

C15orf41 chromosome 10 C15orf41 homolog

C15orf48 chromosome 15 open reading frame 48

C15orf65 chromosome 15 open reading frame 65

C16orf54 chromosome 25 C16orf54 homolog

C16orf58 chromosome 25 C16orf58 homolog

C16orf87 chromosome 18 C16orf87 homolog

C19orf25 chromosome 7 C19orf25 homolog

C1orf87 chromosome 3 C1orf87 homolog

C21orf91 chromosome 1 C21orf91 homolog

C2CD4C C2 calcium dependent domain containing 4C

C2orf50 chromosome 11 C2orf50 homolog

C2orf73 chromosome 11 C2orf73 homolog

**TABLE S 1**: GENES AND GENES DESCRIPTIONS BASED ON REGIONS WITH HIGH FREQUENCY OF ROH (Continued)

**Gene name Gene description**

C3orf14 chromosome 22 C3orf14 homolog

C3orf49 chromosome 3 open reading frame 49

C3orf70 chromosome 1 C3orf70 homolog

C5orf15 chromosome 7 C5orf15 homolog

C5orf24 chromosome 7 C5orf24 homolog

C5orf47 chromosome 5 open reading frame 47

C9orf16 chromosome 11 C9orf16 homolog

CACHD1 cache domain containing 1

CALN1 calneuron 1

CAMK2A calcium/calmodulin dependent protein kinase II alpha

CAMK2N2 calcium/calmodulin dependent protein kinase II inhibitor 2

CAMLG calcium modulating ligand

CASP6 caspase 6

CATSPER3 cation channel sperm associated 3

CAVIN2 caveolae associated protein 2

CCDC189 coiled-coil domain containing 189

CCDC42 coiled-coil domain containing 42

CCDC69 coiled-coil domain containing 69

CCDC70 coiled-coil domain containing 70

CCDC85A coiled-coil domain containing 85A

CCDC88A coiled-coil domain containing 88A

CCN1 cellular communication network factor 1

CCPG1 cell cycle progression 1

CCSER1 coiled-coil serine rich protein 1

CD226 CD226 molecule

CD2BP2 CD2 cytoplasmic tail binding protein 2

CD74 CD74 molecule

CD82 CD82 molecule

CD8A CD8a molecule

CDC23 cell division cycle 23

CDC25C cell division cycle 25C

CDC34 cell division cycle 34

CDIPT CDP-diacylglycerol--inositol 3-phosphatidyltransferase

CDK9 cyclin dependent kinase 9

CDKL3 cyclin dependent kinase like 3

CDKN2AIPNL CDKN2A interacting protein N-terminal like

CDRT4 CMT1A duplicated region transcript 4

CDX1 caudal type homeobox 1

CEP152 centrosomal protein 152

CEP78 centrosomal protein 78

CFAP157 cilia and flagella associated protein 157

CFAP52 cilia and flagella associated protein 52

CFD complement factor D

CGNL1 cingulin like 1

CHCHD5 coiled-coil-helix-coiled-coil-helix domain containing 5

**TABLE S 1**: GENES AND GENES DESCRIPTIONS BASED ON REGIONS WITH HIGH FREQUENCY OF ROH (Continued)

**Gene name Gene description**

CHD9 chromodomain helicase DNA binding protein 9

chi-mir-101 chi-mir-101

chi-mir-125b chi-mir-125b

chi-mir-143 chi-mir-143

chi-mir-145 chi-mir-145

chi-mir-147 chi-mir-147

chi-mir-16a chi-mir-16a

chi-mir-216b chi-mir-216b

chi-mir-33b chi-mir-33b

chi-mir-582 chi-mir-582

chi-mir-99a chi-mir-99a

CHODL chondrolectin

CHRD chordin

CHRM5 cholinergic receptor muscarinic 5

CHRNA10 neuronal acetylcholine receptor subunit alpha-10

CHRNA3 cholinergic receptor nicotinic alpha 3 subunit

CHRNA5 cholinergic receptor nicotinic alpha 5 subunit

CHRNB4 cholinergic receptor nicotinic beta 4 subunit

CIRBP cold inducible RNA binding protein

CIZ1 CDKN1A interacting zinc finger protein 1

CKAP2 cytoskeleton associated protein 2

CKAP2L cytoskeleton associated protein 2 like

CLCA1 chloride channel accessory 1

CLCA2 chloride channel accessory 2

CLCA4 chloride channel accessory 4

CLCN2 chloride voltage-gated channel 2

CLEC3A C-type lectin domain family 3 member A

CLHC1 clathrin heavy chain linker domain containing 1

CLPB ClpB homolog, mitochondrial AAA ATPase chaperonin

COG6 component of oligomeric golgi complex 6

COL15A1 collagen type XV alpha 1 chain

COL24A1 collagen type XXIV alpha 1 chain

COL25A1 collagen type XXV alpha 1 chain

COL6A2 collagen type VI alpha 2 chain

COPB1 coatomer protein complex subunit beta 1

COPS2 COP9 signalosome subunit 2

COPS3 COP9 signalosome subunit 3

CORO1A coronin 1A

COX10 protoheme IX farnesyltransferase, mitochondrial

COX6A2 cytochrome c oxidase subunit 6A2, mitochondrial

CPEB4 cytoplasmic polyadenylation element binding protein 4

CRCP CGRP receptor component

CREBRF CREB3 regulatory factor

CRH corticotropin releasing hormone

CRPPA CDP-L-ribitol pyrophosphorylase A

CRSP-1 calcitonin receptor-stimulating peptide-1

**TABLE S 1**: GENES AND GENES DESCRIPTIONS BASED ON REGIONS WITH HIGH FREQUENCY OF ROH (Continued)

**Gene name Gene description**

CRSP-2 calcitonin receptor-stimulating peptide-2

CSF1R colony stimulating factor 1 receptor

CSNK1A1 casein kinase 1 alpha 1

CSNK1G2 casein kinase 1 gamma 2

CTF1 cardiotrophin 1

CTNNA1 catenin alpha 1

CTXN2 cortexin 2

CXADR CXADR Ig-like cell adhesion molecule

CXCL14 C-X-C motif chemokine ligand 14

CYP2R1 vitamin D 25-hydroxylase

CYP2U1 cytochrome P450 2U1

CYP7B1 25-hydroxycholesterol 7-alpha-hydroxylase

CYS1 cystin 1

DAZAP1 DAZ associated protein 1

DCAF10 DDB1 and CUL4 associated factor 10

DCTN4 dynactin subunit 4

DCTPP1 dCTP pyrophosphatase 1

DDAH1 dimethylarginine dimethylaminohydrolase 1

DDHD1 DDHD domain containing 1

DDX46 DEAD-box helicase 46

DHRS12 dehydrogenase/reductase 12

DHRS7C dehydrogenase/reductase 7C

DIP2A disco interacting protein 2 homolog A

DIPK2A divergent protein kinase domain 2A

DKK2 dickkopf WNT signaling pathway inhibitor 2

DNAAF4 dynein axonemal assembly factor 4

DNAJB4 DnaJ heat shock protein family (Hsp40) member B4

DNAJB5 DnaJ heat shock protein family (Hsp40) member B5

DNAJC5B DnaJ heat shock protein family (Hsp40) member C5 beta

DOC2A double C2 domain alpha

DOCK7 dedicator of cytokinesis 7

DOK4 docking protein 4

DOK6 docking protein 6

DPH6 diphthamine biosynthesis 6

DPM2 dolichyl-phosphate mannosyltransferase subunit 2, regulatory

DPYSL3 dihydropyrimidinase like 3

DRC3 dynein regulatory complex subunit 3

DRG2 developmentally regulated GTP binding protein 2

DTWD1 DTW domain containing 1

DUOX1 dual oxidase 1

DUOX2 dual oxidase 2

DUOXA1 dual oxidase maturation factor 1

DUOXA2 dual oxidase maturation factor 2

DVL3 dishevelled segment polarity protein 3

E2F6 E2F transcription factor 6

**TABLE S 1**: GENES AND GENES DESCRIPTIONS BASED ON REGIONS WITH HIGH FREQUENCY OF ROH (Continued)

**Gene name Gene description**

EBPL EBP like

ECE2 endothelin converting enzyme 2

EFCAB7 EF-hand calcium binding domain 7

EFEMP1 EGF containing fibulin extracellular matrix protein 1

EFNA2 ephrin A2

EGR1 early growth response 1

EHHADH enoyl-CoA hydratase and 3-hydroxyacyl CoA dehydrogenase

EIF2AK3 eukaryotic translation initiation factor 2 alpha kinase 3

EIF2B5 eukaryotic translation initiation factor 2B subunit epsilon

EIF4G1 eukaryotic translation initiation factor 4 gamma 1

ELANE elastase, neutrophil expressed

ELOVL6 ELOVL fatty acid elongase 6

EMC4 ER membrane protein complex subunit 4

EMC7 ER membrane protein complex subunit 7

EML6 EMAP like 6

ENG endoglin

EPHB3 EPH receptor B3

EPN2 epsin 2

ERGIC1 endoplasmic reticulum-golgi intermediate compartment 1

ERP44 endoplasmic reticulum protein 44

ETF1 eukaryotic translation termination factor 1

ETFA electron transfer flavoprotein subunit alpha

ETNPPL ethanolamine-phosphate phospho-lyase

EXOSC3 exosome component 3

EXT2 exostosin glycosyltransferase 2

FABP1 fatty acid binding protein 1

FAM102A family with sequence similarity 102 member A

FAM124A family with sequence similarity 124 member A

FAM131A family with sequence similarity 131 member A

FAM13B family with sequence similarity 13 member B

FAM151B family with sequence similarity 151 member B

FAM174C family with sequence similarity 174 member C

FAM214B family with sequence similarity 214 member B

FAM53C family with sequence similarity 53 member C

FAM83G family with sequence similarity 83 member G

FANCG FA complementation group G

FAT2 FAT atypical cadherin 2

FBLL1 fibrillarin like 1

FBN1 fibrillin 1

FBXL19 F-box and leucine rich repeat protein 19

FBXO10 F-box protein 10

FBXO22 F-box protein 22

FBXO25 F-box protein 25

FBXO38 F-box protein 38

FBXO5 F-box protein 5

**TABLE S 1**: GENES AND GENES DESCRIPTIONS BASED ON REGIONS WITH HIGH FREQUENCY OF ROH (Continued)

**Gene name Gene description**

FERMT2 fermitin family member 2

FEZF2 FEZ family zinc finger 2

FGF22 fibroblast growth factor 22

FGF7 fibroblast growth factor 7

FGGY FGGY carbohydrate kinase domain containing

FKTN fukutin

FLII FLII actin remodeling protein

FMN1 formin 1

FOXE1 forkhead box E1

FOXI3 forkhead box I3

FOXO1 forkhead box O1

FPGS folylpolyglutamate synthase

FRMPD1 FERM and PDZ domain containing 1

FRY FRY microtubule binding protein

FSD1L fibronectin type III and SPRY domain containing 1 like

FSTL3 follistatin like 3

FSTL4 follistatin like 4

FSTL5 follistatin like 5

FTCD formimidoyltransferase cyclodeaminase

FTO FTO alpha-ketoglutarate dependent dioxygenase

FUS FUS RNA binding protein

G3BP1 G3BP stress granule assembly factor 1

GABBR2 gamma-aminobutyric acid type B receptor subunit 2

GABPB1 GA binding protein transcription factor subunit beta 1

GALK2 galactokinase 2

GALNT12 polypeptide N-acetylgalactosaminyltransferase 12

GALNT17 polypeptide N-acetylgalactosaminyltransferase 17

GAMT guanidinoacetate N-methyltransferase

GAPT GRB2 binding adaptor protein, transmembrane

GAPVD1 GTPase activating protein and VPS9 domains 1

GARNL3 GTPase activating Rap/RanGAP domain like 3

GATM glycine amidinotransferase

GDF9 growth differentiation factor 9

GDPD3 glycerophosphodiester phosphodiesterase domain containing 3

GFRA3 GDNF family receptor alpha 3

GID4 GID complex subunit 4 homolog

GIPC2 GIPC PDZ domain containing family member 2

GLB1L3 beta-galactosidase-1-like protein 3

GLP2R glucagon like peptide 2 receptor

GLRA1 glycine receptor alpha 1

GM2A GM2 ganglioside activator

GPBP1 GC-rich promoter binding protein 1

GPT2 glutamic--pyruvic transaminase 2

GPX3 glutathione peroxidase 3

GPX4 glutathione peroxidase 4

**TABLE S 1**: GENES AND GENES DESCRIPTIONS BASED ON REGIONS WITH HIGH FREQUENCY OF ROH (Continued)

**Gene name Gene description**

GREB1 growth regulating estrogen receptor binding 1

GRHPR glyoxylate and hydroxypyruvate reductase

GRIA1 glutamate ionotropic receptor AMPA type subunit 1

GRID2 glutamate ionotropic receptor delta type subunit 2

GRIN3B glutamate ionotropic receptor NMDA type subunit 3B

GRPEL2 GrpE like 2, mitochondrial

HADH hydroxyacyl-CoA dehydrogenase

HCN2 hyperpolarization activated cyclic nucleotide gated potassium and sodium

channel 2

HDC histidine decarboxylase

HEMGN hemogen

HIRIP3 HIRA interacting protein 3

HMGXB3 HMG-box containing 3

HNRNPA0 heterogeneous nuclear ribonucleoprotein A0

HOOK1 hook microtubule tethering protein 1

HPCAL1 hippocalcin like 1

HS3ST3B1 heparan sulfate glucosamine 3-O-sulfotransferase 3B1

HSD17B12 hydroxysteroid 17-beta dehydrogenase 12

HSD3B7 hydroxy-delta-5-steroid dehydrogenase, 3 beta- and steroid

delta-isomerase 7

HSPA5 heat shock protein family A (Hsp70) member 5

HSPA9 heat shock protein family A (Hsp70) member 9

HTR4 5-hydroxytryptamine receptor 4

HYKK hydroxylysine kinase

IGFBPL1 insulin like growth factor binding protein like 1

IL17B interleukin 17B

IL18BP interleukin 18 binding protein

IL1A interleukin 1 alpha

IL1B interleukin 1 beta

IL1F10 interleukin 1 family member 10

IL1RN interleukin 1 receptor antagonist

IL27 interleukin 27

IL36A interleukin-36 alpha

IL36G interleukin-36 gamma

IL36RN interleukin 36 receptor antagonist

IMMT inner membrane mitochondrial protein

INO80E INO80 complex subunit E

INPPL1 inositol polyphosphate phosphatase like 1

INSC INSC spindle orientation adaptor protein

INTS6 integrator complex subunit 6

INVS inversin

IPCEF1 interaction protein for cytohesin exchange factors 1

IREB2 iron responsive element binding protein 2

IRX3 iroquois homeobox 3

IRX5 iroquois homeobox 5

ISL2 ISL LIM homeobox 2

**TABLE S 1**: GENES AND GENES DESCRIPTIONS BASED ON REGIONS WITH HIGH FREQUENCY OF ROH (Continued)

**Gene name Gene description**

ITGAD integrin alpha-D

ITGAL integrin subunit alpha L

ITGAM integrin subunit alpha M

JADE2 jade family PHD finger 2

JAK1 Janus kinase 1

JAKMIP2 janus kinase and microtubule interacting protein 2

KANK4 KN motif and ankyrin repeat domains 4

KAT8 lysine acetyltransferase 8

KCNF1 potassium voltage-gated channel modifier subfamily F member 1

KCNRG potassium channel regulator

KCNT2 potassium sodium-activated channel subfamily T member 2

KCTD13 potassium channel tetramerization domain containing 13

KCTD7 potassium channel tetramerization domain containing 7

KDM3A lysine demethylase 3A

KDM3B lysine demethylase 3B

KHDRBS3 KH RNA binding domain containing, signal transduction associated 3

KIF20A kinesin family member 20A

KIF22 kinesin family member 22

KL klotho

KLF11 Kruppel like factor 11

KLF16 Kruppel like factor 16

KLF4 Kruppel like factor 4

KLHL24 kelch like family member 24

KLHL3 kelch like family member 3

KPNA3 karyopherin subunit alpha 3

KRCC1 lysine rich coiled-coil 1

LAMTOR1 late endosomal/lysosomal adaptor, MAPK and MTOR activator 1

LCN2 lipocalin 2

LEAP2 liver enriched antimicrobial peptide 2

LECT2 leukocyte cell derived chemotaxin 2

LEF1 lymphoid enhancer binding factor 1

LHFPL6 LHFPL tetraspan subfamily member 6

LIPC lipase C, hepatic type

LIPH lipase H

LLGL1 LLGL scribble cell polarity complex component 1

LMX1B LIM homeobox transcription factor 1 beta

LPCAT4 lysophosphatidylcholine acyltransferase 4

LPIN1 lipin 1

LRIT3 leucine rich repeat, Ig-like and transmembrane domains 3

LRRC4C leucine rich repeat containing 4C

LRRC72 leucine rich repeat containing 72

LRRC75A leucine rich repeat containing 75A

LRRTM2 leucine rich repeat transmembrane neuronal 2

LRSAM1 leucine rich repeat and sterile alpha motif containing 1

LSS lanosterol synthase

**TABLE S 1**: GENES AND GENES DESCRIPTIONS BASED ON REGIONS WITH HIGH FREQUENCY OF ROH (Continued)

**Gene name Gene description**

MAB21L1 mab-21 like 1

MACROH2A1 core histone macro-H2A.1

MAF MAF bZIP transcription factor

MAGEF1 MAGE family member F1

MAGI1 membrane associated guanylate kinase, WW and PDZ domain

containing 1

MAP3K1 mitogen-activated protein kinase kinase kinase 1

MAP3K13 mitogen-activated protein kinase kinase kinase 13

MAP6D1 MAP6 domain containing 1

MAPK3 mitogen-activated protein kinase 3

MAPK7 mitogen-activated protein kinase 7

MAPKAP1 MAPK associated protein 1

MAZ MYC associated zinc finger protein

MBD3 methyl-CpG binding domain protein 3

MCF2L2 MCF.2 cell line derived transforming sequence-like 2

MCM3AP minichromosome maintenance complex component 3 associated protein

MCOLN2 mucolipin 2

MCOLN3 mucolipin 3

MCUB mitochondrial calcium uniporter dominant negative beta subunit

MED16 mediator complex subunit 16

MED9 mediator complex subunit 9

MEIS2 Meis homeobox 2

MELK maternal embryonic leucine zipper kinase

MEOX2 mesenchyme homeobox 2

Metazoa_SRP Metazoan signal recognition particle RNA

MEX3D mex-3 RNA binding family member D

MFAP4 microfibril associated protein 4

MFSD6L major facilitator superfamily domain containing 6 like

MIDN midnolin

MIEF2 mitochondrial elongation factor 2

MIER2 MIER family member 2

MIER3 MIER family member 3

MIGA1 mitoguardin 1

MIR1224 microRNA mir-1224

MIR129 microRNA mir-129

MIR15A microRNA mir-15a

MIR216A microRNA 216a

MIR30D microRNA mir-30d

MIR378 microRNA mir-378

MIR628 microRNA mir-628

MIR874 microRNA mir-874

MIRLET7C microRNA let-7c

MISP mitotic spindle positioning

MNS1 meiosis specific nuclear structural 1

MRPL35 mitochondrial ribosomal protein L35

MRPS31 mitochondrial ribosomal protein S31

**TABLE S 1**: GENES AND GENES DESCRIPTIONS BASED ON REGIONS WITH HIGH FREQUENCY OF ROH (Continued)

**Gene name Gene description**

MSH3 mutS homolog 3

MTFR1 mitochondrial fission regulator 1

MTIF2 mitochondrial translational initiation factor 2

MTRF1L mitochondrial translational release factor 1 like

MVB12B multivesicular body subunit 12B

MVP major vault protein

MYBL1 MYB proto-oncogene like 1

MYEF2 myelin expression factor 2

MYH10 myosin heavy chain 10

MYH2 myosin heavy chain 2

MYH3 myosin-3

MYH4 myosin-4

MYH8 myosin heavy chain 8

MYLK3 myosin light chain kinase 3

MYLPF myosin light chain, phosphorylatable, fast skeletal muscle

MYO15A myosin XVA

MYO1B myosin IB

MYOT myotilin

MYOZ3 myozenin 3

N4BP2L1 NEDD4 binding protein 2 like 1

NABP1 nucleic acid binding protein 1

NAIF1 nuclear apoptosis inducing factor 1

NANS N-acetylneuraminate synthase

NBEA neurobeachin

NCBP1 nuclear cap binding protein subunit 1

NCOR1 nuclear receptor corepressor 1

NDST1 N-deacetylase and N-sulfotransferase 1

NDUFS7 NADH:ubiquinone oxidoreductase core subunit S7

NEDD4 NEDD4 E3 ubiquitin protein ligase

NEK3 NIMA related kinase 3

NEK5 NIMA related kinase 5

NETO2 neuropilin and tolloid like 2

NEUROG1 neurogenin 1

NEXN nexilin F-actin binding protein

NFIA nuclear factor I A

NIBAN2 niban apoptosis regulator 2

NKX2-5 NK2 homeobox 5

NKX3-2 NK3 homeobox 2

NME5 NME/NM23 family member 5

NMUR2 neuromedin U receptor 2

NOL10 nucleolar protein 10

NOP10 NOP10 ribonucleoprotein

NR4A3 nuclear receptor subfamily 4 group A member 3

NRG4 neuregulin 4

NRON non-coding repressor of NFAT

**TABLE S 1**: GENES AND GENES DESCRIPTIONS BASED ON REGIONS WITH HIGH FREQUENCY OF ROH (Continued)

**Gene name Gene description**

NSG2 neuronal vesicle trafficking associated 2

NT5DC4 5'-nucleotidase domain containing 4

NT5M 5',3'-nucleotidase, mitochondrial

NTN1 netrin 1

NTSR2 neurotensin receptor 2

NUDT7 nudix hydrolase 7

NUMA1 nuclear mitotic apparatus protein 1

NUP98 nucleoporin 98

NUPR1 nuclear protein 1, transcriptional regulator

NUPR2 nuclear protein 2, transcriptional regulator

ODF2L outer dense fiber of sperm tails 2 like

ODF3L2 outer dense fiber of sperm tails 3 like 2

OPRM1 opioid receptor mu 1

OR4K15 olfactory receptor family 4 subfamily K member 15

OR4N5 olfactory receptor 4N5

ORAI3 ORAI calcium release-activated calcium modulator 3

ORC6 origin recognition complex subunit 6

PAGR1 PAXIP1 associated glutamate rich protein 1

PALM paralemmin

PAPSS1 3'-phosphoadenosine 5'-phosphosulfate synthase 1

PATJ PATJ crumbs cell polarity complex component

PAX5 paired box 5

PAX8 paired box 8

PBX3 PBX homeobox 3

PCBD2 pterin-4 alpha-carbinolamine dehydratase 2

PCBP3 poly(rC) binding protein 3

PCSK4 proprotein convertase subtilisin/kexin type 4

PCYOX1L prenylcysteine oxidase 1 like

PDE2A phosphodiesterase 2A

PDE3B phosphodiesterase 3B

PDE4D phosphodiesterase 4D

PDE6A phosphodiesterase 6A

PDE7A phosphodiesterase 7A

PDGFRB platelet derived growth factor receptor beta

PDIA6 protein disulfide isomerase family A member 6

PDS5B PDS5 cohesin associated factor B

PEAK1 pseudopodium enriched atypical kinase 1

PEMT phosphatidylethanolamine N-methyltransferase

PGAP2 post-GPI attachment to proteins 2

PGBD2 piggyBac transposable element derived 2

PGM1 phosphoglucomutase 1

PHF24 PHD finger protein 24

PHKB phosphorylase kinase regulatory subunit beta

PHKG1 phosphorylase kinase catalytic subunit gamma 1

PHKG2 phosphorylase kinase catalytic subunit gamma 2

**TABLE S 1**: GENES AND GENES DESCRIPTIONS BASED ON REGIONS WITH HIGH FREQUENCY OF ROH (Continued)

**Gene name Gene description**

PHOX2A paired like homeobox 2A

PIGB phosphatidylinositol glycan anchor biosynthesis class B

PIGL phosphatidylinositol glycan anchor biosynthesis class L

PIGO phosphatidylinositol glycan anchor biosynthesis class O

PIK3R5 phosphoinositide-3-kinase regulatory subunit 5

PIK3R6 phosphoinositide-3-kinase regulatory subunit 6

PIP5KL1 phosphatidylinositol-4-phosphate 5-kinase like 1

PITX1 paired like homeodomain 1

PKD2L2 polycystin 2 like 2, transient receptor potential cation channel

PLA2G12A phospholipase A2 group XIIA

PLD6 phospholipase D family member 6

PLK2 polo like kinase 2

PLPP2 phospholipid phosphatase 2

PLPPR3 phospholipid phosphatase related 3

PMP22 peripheral myelin protein 22

PNPT1 polyribonucleotide nucleotidyltransferase 1

POFUT2 protein O-fucosyltransferase 2

POLR1A RNA polymerase I subunit A

POLR1B RNA polymerase I subunit B

POLR1E RNA polymerase I subunit E

POLR2C RNA polymerase II subunit C

POLR2E RNA polymerase II subunit E

POLR2H RNA polymerase II subunit H

POLR2M RNA polymerase II subunit M

POLRMT RNA polymerase mitochondrial

PPARGC1B PPARG coactivator 1 beta

PPP2CA protein phosphatase 2 catalytic subunit alpha

PPP4C protein phosphatase 4 catalytic subunit

PPP4R3B protein phosphatase 4 regulatory subunit 3B

PPP6C protein phosphatase 6 catalytic subunit

PRICKLE2 prickle planar cell polarity protein 2

PRMT2 protein arginine methyltransferase 2

PRPSAP2 phosphoribosyl pyrophosphate synthetase associated protein 2

PRR14 proline rich 14

PRSS36 serine protease 36

PRSS53 serine protease 53

PRSS57 serine protease 57

PRSS8 serine protease 8

PRTG protogenin

PRTN3 proteinase 3

PSAT1 phosphoserine aminotransferase 1

PSD4 pleckstrin and Sec7 domain containing 4

PSMA1 proteasome 20S subunit alpha 1

PSMA4 proteasome 20S subunit alpha 4

PSMD2 proteasome 26S subunit, non-ATPase 2

**TABLE S 1**: GENES AND GENES DESCRIPTIONS BASED ON REGIONS WITH HIGH FREQUENCY OF ROH (Continued)

**Gene name Gene description**

PSMD6 proteasome 26S subunit, non-ATPase 6

PSPH phosphoserine phosphatase

PSTPIP1 proline-serine-threonine phosphatase interacting protein 1

PTBP1 polypyrimidine tract binding protein 1

PTCD3 pentatricopeptide repeat domain 3

PTGES2 prostaglandin E synthase 2

PTGFR prostaglandin F receptor

PTPRG protein tyrosine phosphatase receptor type G

PTRH1 peptidyl-tRNA hydrolase 1 homolog

PWWP3A PWWP domain containing 3A, DNA repair factor

PYCARD apoptosis-associated speck-like protein containing a CARD

PYGO1 pygopus family PHD finger 1

QPRT quinolinate phosphoribosyltransferase

R3HDM4 R3H domain containing 4

RAB28 RAB28, member RAS oncogene family

RAB3C RAB3C, member RAS oncogene family

RABEPK Rab9 effector protein with kelch motifs

RAD23B RAD23 homolog B, nucleotide excision repair protein

RAI1 retinoic acid induced 1

RALGPS1 Ral GEF with PH domain and SH3 binding motif 1

RARS1 arginyl-tRNA synthetase 1

RASD1 ras related dexamethasone induced 1

RASGRF2 Ras protein specific guanine nucleotide releasing factor 2

RAVER2 ribonucleoprotein, PTB binding 2

RBL2 RB transcriptional corepressor like 2

RBM22 RNA binding motif protein 22

RCBTB1 RCC1 and BTB domain containing protein 1

RCN2 reticulocalbin 2

RCVRN recoverin

REEP1 receptor accessory protein 1

REEP2 receptor accessory protein 2

REEP6 receptor accessory protein 6

REXO1 RNA exonuclease 1 homolog

RFC3 replication factor C subunit 3

RFX7 regulatory factor X7

RGS17 regulator of G protein signaling 17

RHOG ras homolog family member G

RMND5A required for meiotic nuclear division 5 homolog A

RNASEH2B ribonuclease H2 subunit B

RNF103 charged multivesicular body protein 3

RNF112 ring finger protein 112

RNF121 ring finger protein 121

RNF126 ring finger protein 126

RNF38 ring finger protein 38

RNF40 ring finger protein 40

**TABLE S 1**: GENES AND GENES DESCRIPTIONS BASED ON REGIONS WITH HIGH FREQUENCY OF ROH (Continued)

**Gene name Gene description**

ROCK2 Rho associated coiled-coil containing protein kinase 2

ROR1 receptor tyrosine kinase like orphan receptor 1

RPGRIP1L RPGRIP1 like

RPIA ribose 5-phosphate isomerase A

RPL12 ribosomal protein L12

RPL26L1 ribosomal protein L26 like 1

RRAS2 RAS related 2

RRH retinal pigment epithelium-derived rhodopsin homolog

RRM2 ribonucleotide reductase regulatory subunit M2

RRS1 ribosome biogenesis regulator 1 homolog

RTN4 reticulon 4

RTTN rotatin

RUNX1 RUNX family transcription factor 1

RYR3 ryanodine receptor 3

S100B S100 calcium binding protein B

SAR1B secretion associated Ras related GTPase 1B

SBNO2 strawberry notch homolog 2

SCAMP4 secretory carrier membrane protein 4

SCAPER S-phase cyclin A associated protein in the ER

SCGB3A2 secretoglobin family 3A member 2

SEC24A SEC24 homolog A, COPII coat complex component

SEC24B SEC24 homolog B, COPII coat complex component

SECISBP2L SECIS binding protein 2 like

SELENOF selenoprotein F

SEMA6D semaphorin 6D

SENP2 SUMO specific peptidase 2

SEPTIN1 septin 1

SEPTIN14 septin 14

SERPINE3 serpin family E member 3

SETD1A SET domain containing 1A, histone lysine methyltransferase

SETD9 SET domain containing 9

SETDB2 SET domain bifurcated histone lysine methyltransferase 2

SEZ6L2 seizure related 6 homolog like 2

SGF29 SAGA complex associated factor 29

SGMS2 sphingomyelin synthase 2

SH2D3C SH2 domain containing 3C

SH3BP5L SH3 binding domain protein 5 like

SH3GLB1 SH3 domain containing GRB2 like, endophilin B1

SH3TC2 SH3 domain and tetratricopeptide repeats 2

SHC4 SHC adaptor protein 4

SHCBP1 SHC binding and spindle associated 1

SHF Src homology 2 domain containing F

SHMT1 serine hydroxymethyltransferase 1

SHROOM1 shroom family member 1

SIL1 SIL1 nucleotide exchange factor

**TABLE S 1**: GENES AND GENES DESCRIPTIONS BASED ON REGIONS WITH HIGH FREQUENCY OF ROH (Continued)

**Gene name Gene description**

SKP1 S-phase kinase associated protein 1

SLC12A1 solute carrier family 12 member 1

SLC12A6 solute carrier family 12 member 6

SLC19A1 solute carrier family 19 member 1

SLC20A1 solute carrier family 20 member 1

SLC24A5 solute carrier family 24 member 5

SLC25A15 solute carrier family 25 member 15

SLC25A25 solute carrier family 25 member 25

SLC25A48 solute carrier family 25 member 48

SLC26A2 solute carrier family 26 member 2

SLC27A2 solute carrier family 27 member 2

SLC28A2 solute carrier family 28 member 2

SLC2A8 solute carrier family 2 member 8

SLC30A4 solute carrier family 30 member 4

SLC36A1 proton-coupled amino acid transporter 1

SLC36A2 solute carrier family 36 member 2

SLC36A3 proton-coupled amino acid transporter 3

SLC44A1 solute carrier family 44 member 1

SLC47A1 solute carrier family 47 member 1

SLC47A2 solute carrier family 47 member 2

SLC5A10 solute carrier family 5 member 10

SLC5A2 solute carrier family 5 member 2

SLC66A3 solute carrier family 66 member 3

SLC6A7 solute carrier family 6 member 7

SLC9A9 solute carrier family 9 member A9

SMAD5 SMAD family member 5

SMCR8 SMCR8-C9orf72 complex subunit

SMIM3 small integral membrane protein 3

SMIM32 small integral membrane protein 32

SMYD1 SET and MYND domain containing 1

SNORA62 small nucleolar RNA SNORA62/SNORA6 family

SNORA63 small nucleolar RNA SNORA63

SNORA65 small nucleolar RNA, H/ACA box 65

SNORA70 small nucleolar RNA SNORA70

SNORA71 small nucleolar RNA SNORA71

SNORA72 small nucleolar RNA SNORA72

SNORA80B small nucleolar RNA, H/ACA box 80B

SNORD14 small nucleolar RNA SNORD14

SNORD31 small nucleolar RNA SNORD31

SNORD49A small nucleolar RNA, C/D box 49A

SNORD49B small nucleolar RNA, C/D box 49B

SNORD63 small nucleolar RNA SNORD63

SNORD65 small nucleolar RNA, C/D box 65

SNORD66 small nucleolar RNA, C/D box 66

SNORD94 small nucleolar RNA, C/D box 94

**TABLE S 1**: GENES AND GENES DESCRIPTIONS BASED ON REGIONS WITH HIGH FREQUENCY OF ROH (Continued)

**Gene name Gene description**

SNTN sentan, cilia apical structure protein

SORD sorbitol dehydrogenase

SOSTDC1 sclerostin domain containing 1

SOX6 SRY-box transcription factor 6

SPARC secreted protein acidic and cysteine rich

SPATA5L1 spermatogenesis associated 5 like 1

SPATC1L spermatogenesis and centriole associated 1 like

SPECC1 cytospin-B

SPINK1 serine peptidase inhibitor, Kazal type

SPINK5 serine protease inhibitor Kazal-type 5

SPINK6 serine protease inhibitor Kazal-type 6

SPINK7 serine peptidase inhibitor, Kazal type 7 (putative)

SPINK9 serine peptidase inhibitor, Kazal type 9

SPOCK1 SPARC (osteonectin), cwcv and kazal like domains proteoglycan 1

SPRYD7 SPRY domain containing 7

SPTBN1 spectrin beta, non-erythrocytic 1

ST3GAL1 ST3 beta-galactoside alpha-2,3-sialyltransferase 1

ST6GALNAC4 ST6 N-acetylgalactosaminide alpha-2,6-sialyltransferase 4

STARD13 StAR related lipid transfer domain containing 13

STC2 stanniocalcin 2

STIM1 stromal interaction molecule 1

STK11 serine/threonine kinase 11

STOML2 stomatin like 2

STX17 syntaxin 17

STX1B syntaxin 1B

STX4 syntaxin 4

STX8 syntaxin 8

STXBP1 syntaxin binding protein 1

SYDE2 synapse defective Rho GTPase homolog 2

SYNPO synaptopodin

SYNPR synaptoporin

TAF1B TATA-box binding protein associated factor, RNA polymerase I

subunit B

TAL2 TAL bHLH transcription factor 2

TAOK2 TAO kinase 2

TBC1D10B TBC1 domain family member 10B

TBC1D2 TBC1 domain family member 2

TBX6 T-box transcription factor 6

TCF12 transcription factor 12

TCF3 transcription factor 3

TCF7 transcription factor 7

TCOF1 treacle ribosome biogenesis factor 1

TDRD7 tudor domain containing 7

TDRP testis development related protein

TEKT3 tektin 3

TENM2 teneurin-2

**TABLE S 1**: GENES AND GENES DESCRIPTIONS BASED ON REGIONS WITH HIGH FREQUENCY OF ROH (Continued)

**Gene name Gene description**

TEX37 testis expressed 37

TEX9 testis expressed 9

TGFB1I1 transforming growth factor beta 1 induced transcript 1

TGFBI transforming growth factor beta induced

TGFBR1 transforming growth factor beta receptor 1

THEG theg spermatid protein

THNSL2 threonine synthase like 2

THOC7 THO complex 7

THPO thrombopoietin

THSD1 thrombospondin type 1 domain containing 1

TIFAB TIFA inhibitor

TIGD6 tigger transposable element derived 6

TLCD3B TLC domain containing 3B

TLE1 transducin-like enhancer protein 1

TM2D1 TM2 domain containing 1

TMEFF2 transmembrane protein with EGF like and two follistatin like domains 2

TMEM259 transmembrane protein 259

TMEM266 transmembrane protein 266

TMEM38B transmembrane protein 38B

TMOD1 tropomodulin 1

TMPRSS15 transmembrane serine protease 15

TMX3 thioredoxin related transmembrane protein 3

TNIP1 TNFAIP3 interacting protein 1

TOM1L2 target of myb1 like 2 membrane trafficking protein

TOP3A DNA topoisomerase III alpha

TOR2A torsin family 2 member A

TP53I11 tumor protein p53 inducible protein 11

TPGS1 tubulin polyglutamylase complex subunit 1

TPST1 tyrosylprotein sulfotransferase 1

TRIM13 tripartite motif containing 13

TRIM14 tripartite motif containing 14

TRIM55 tripartite motif containing 55

TRIM72 tripartite motif containing 72

TRMO tRNA methyltransferase O

TRMT10B tRNA methyltransferase 10B

TRPC3 transient receptor potential cation channel subfamily C member 3

TRPC7 transient receptor potential cation channel subfamily C member 7

TRPV2 transient receptor potential cation channel subfamily V member 2

TSPAN18 tetraspanin 18

TSPAN3 tetraspanin 3

TSTD2 thiosulfate sulfurtransferase like domain containing 2

TTC16 tetratricopeptide repeat domain 16

TTC17 tetratricopeptide repeat domain 17

TTC19 tetratricopeptide repeat domain 19

TTL tubulin tyrosine ligase

**TABLE S 1**: GENES AND GENES DESCRIPTIONS BASED ON REGIONS WITH HIGH FREQUENCY OF ROH (Continued)

**Gene name Gene description**

TXNDC15 thioredoxin domain containing 15

U1 U1 spliceosomal RNA

U2 U2 spliceosomal RNA

U4 U4 spliceosomal RNA

U5 U5 spliceosomal RNA

U6 U6 spliceosomal RNA

U8 U8 small nucleolar RNA

UBB ubiquitin B

UBE2B ubiquitin conjugating enzyme E2 B

UBE2Q2 ubiquitin conjugating enzyme E2 Q2

UBE2U ubiquitin conjugating enzyme E2 U

ULK2 unc-51 like autophagy activating kinase 2

UQCRQ cytochrome b-c1 complex subunit 8

USP1 ubiquitin specific peptidase 1

USP25 ubiquitin specific peptidase 25

USP43 ubiquitin specific peptidase 43

USP8 ubiquitin specific peptidase 8

VAT1L vesicle amine transport 1 like

VCP valosin containing protein

VCPIP1 valosin containing protein interacting protein 1

VDAC1 voltage dependent anion channel 1

VKORC1L1 vitamin K epoxide reductase complex subunit 1 like 1

VPS35 VPS35 retromer complex component

VPS36 vacuolar protein sorting 36 homolog

VPS8 VPS8 subunit of CORVET complex

VWA5B2 von Willebrand factor A domain containing 5B2

VXN vexin

WDFY2 WD repeat and FYVE domain containing 2

WDR18 WD repeat domain 18

WDR63 WD repeat domain 63

WNT8A Wnt family member 8A

WWC1 WW and C2 domain containing 1

XPA XPA, DNA damage recognition and repair factor

YBEY ybeY metalloendoribonuclease

YEATS2 YEATS domain containing 2

YPEL3 yippee like 3

ZAR1L zygote arrest 1 like

ZBTB34 zinc finger and BTB domain containing 34

ZBTB43 zinc finger and BTB domain containing 43

ZBTB5 zinc finger and BTB domain containing 5

ZCCHC10 zinc finger CCHC-type containing 10

ZCCHC7 zinc finger CCHC-type containing 7

ZFAT zinc finger and AT-hook domain containing

ZFYVE16 zinc finger FYVE-type containing 16

ZG16 zymogen granule protein 16

**TABLE S 1**: GENES AND GENES DESCRIPTIONS BASED ON REGIONS WITH HIGH FREQUENCY OF ROH (Continued)

**Gene name Gene description**

ZNF287 zinc finger protein 287

ZNF300 zinc finger protein 300

ZNF462 zinc finger protein 462

ZNF48 zinc finger protein 48

ZNF624 zinc finger protein 624

ZNF646 zinc finger protein 646

ZNF668 zinc finger protein 668

ZNF672 zinc finger protein 672

ZNF689 zinc finger protein 689

ZNF692 zinc finger protein 692

ZNF768 zinc finger protein 768

ZNF771 zinc finger protein 771

ZNF79 zinc finger protein 79

ZNHIT6 zinc finger HIT-type containing 6

ZSWIM7 zinc finger SWIM-type containing 7

**TABLE S 2**: KEGG PATHWAYS OF GENES IN THE ROH ISLANDS FOR FREE STATE AND GAUTENG POPULATIONS

**KEGG ID KEGG Pathways**

chx00010 Glycolysis / Gluconeogenesis

chx00030 Pentose phosphate pathway

chx00040 Pentose and glucuronate interconversions

chx00051 Fructose and mannose metabolism

chx00052 Galactose metabolism

chx00062 Fatty acid elongation

chx00071 Fatty acid degradation

chx00100 Steroid biosynthesis

chx00120 Primary bile acid biosynthesis

chx00130 Ubiquinone and other terpenoid-quinone biosynthesis

chx00140 Steroid hormone biosynthesis

chx00190 Oxidative phosphorylation

chx00220 Arginine biosynthesis

chx00230 Purine metabolism

chx00240 Pyrimidine metabolism

chx00250 Alanine

chx00260 Glycine

chx00261 Monobactam biosynthesis

chx00280 Valine

chx00310 Lysine degradation

chx00330 Arginine and proline metabolism

chx00340 Histidine metabolism

chx00350 Tyrosine metabolism

chx00360 Phenylalanine metabolism

chx00380 Tryptophan metabolism

chx00410 beta-Alanine metabolism

chx00450 Selenocompound metabolism

chx00480 Glutathione metabolism

chx00500 Starch and sucrose metabolism

chx00510 N-Glycan biosynthesis

chx00512 Mucin type O-glycan biosynthesis

chx00513 Various types of N-glycan biosynthesis

chx00514 Other types of O-glycan biosynthesis

chx00515 Mannose type O-glycan biosynthesis

chx00520 Amino sugar and nucleotide sugar metabolism

chx00533 Glycosaminoglycan biosynthesiskeratan sulfate

chx00534 Glycosaminoglycan biosynthesisheparan sulfate / heparin

chx00561 Glycerolipid metabolism

chx00562 Inositol phosphate metabolism

chx00563 Glycosylphosphatidylinositol (GPI)-anchor biosynthesis

chx00564 Glycerophospholipid metabolism

chx00565 Ether lipid metabolism

chx00590 Arachidonic acid metabolism

chx00591 Linoleic acid metabolism

chx00592 alpha-Linolenic acid metabolism

**TABLE S 2**: KEGG PATHWAYS OF GENES IN THE ROH ISLANDS FOR FREE STATE AND GAUTENG POPULATIONS (Continued)

**KEGG ID KEGG Pathways**

chx00600 Sphingolipid metabolism

chx00601 Glycosphingolipid biosynthesislacto and neolacto series

chx00603 Glycosphingolipid biosynthesisglobo and isoglobo series

chx00604 Glycosphingolipid biosynthesisganglio series

chx00620 Pyruvate metabolism

chx00630 Glyoxylate and dicarboxylate metabolism

chx00640 Propanoate metabolism

chx00650 Butanoate metabolism

chx00670 One carbon pool by folate

chx00730 Thiamine metabolism

chx00750 Vitamin B6 metabolism

chx00760 Nicotinate and nicotinamide metabolism

chx00790 Folate biosynthesis

chx00830 Retinol metabolism

chx00920 Sulfur metabolism

chx00980 Metabolism of xenobiotics by cytochrome P450

chx00982 Drug metabolismcytochrome P450

chx00983 Drug metabolismother enzymes

chx01040 Biosynthesis of unsaturated fatty acids

chx01100 Metabolic pathways

chx01210 2-Oxocarboxylic acid metabolism

chx01212 Fatty acid metabolism

chx01230 Biosynthesis of amino acids

chx01521 EGFR tyrosine kinase inhibitor resistance

chx01522 Endocrine resistance

chx01523 Antifolate resistance

chx01524 Platinum drug resistance

chx02010 AB

chx03008 Ribosome biogenesis in eukaryotes

chx03010 Ribosome

chx03013 RNA transport

chx03015 mRNA surveillance pathway

chx03018 RNA degradation

chx03020 RNA polymerase

chx03030 DNA replication

chx03040 Spliceosome

chx03050 Proteasome

chx03060 Protein export

chx03320 PPAR signaling pathway

chx03420 Nucleotide excision repair

chx03430 Mismatch repair

chx03440 Homologous recombination

chx03460 Fanconi anemia pathway

chx04010 MAPK signaling pathway

chx04012 ErbB signaling pathway

**TABLE S 2**: KEGG PATHWAYS OF GENES IN THE ROH ISLANDS FOR FREE STATE AND GAUTENG POPULATIONS (Continued)

**KEGG ID KEGG Pathways**

chx04014 Ras signaling pathway

chx04015 Rap1 signaling pathway

chx04022 cGMP-PKG signaling pathway

chx04024 cAMP signaling pathway

chx04061 Viral protein interaction with cytokine and cytokine receptor

chx04064 NF-kappa B signaling pathway

chx04066 HIF-1 signaling pathway

chx04068 FoxO signaling pathway

chx04070 Phosphatidylinositol signaling system

chx04071 Sphingolipid signaling pathway

chx04072 Phospholipase D signaling pathway

chx04080 Neuroactive ligand-receptor interaction

chx04114 Oocyte meiosis

chx04115 p53 signaling pathway

chx04120 Ubiquitin mediated proteolysis

chx04130 SNARE interactions in vesicular transport

chx04136 Autophagyother

chx04137 Mitophagyanimal

chx04140 Autophagyanimal

chx04141 Protein processing in endoplasmic reticulum

chx04142 Lysosome

chx04144 Endocytosis

chx04145 Phagosome

chx04146 Peroxisome

chx04150 mTOR signaling pathway

chx04151 PI3K-Akt signaling pathway

chx04152 AMPK signaling pathway

chx04210 Apoptosis

chx04211 Longevity regulating pathway

chx04213 Longevity regulating pathwaymultiple species

chx04216 Ferroptosis

chx04217 Necroptosis

chx04261 Adrenergic signaling in cardiomyocytes

chx04270 Vascular smooth muscle contraction

chx04310 Wnt signaling pathway

chx04330 Notch signaling pathway

chx04340 Hedgehog signaling pathway

chx04350 TGF-beta signaling pathway

chx04360 Axon guidance

chx04370 VEGF signaling pathway

chx04371 Apelin signaling pathway

chx04380 Osteoclast differentiation

chx04390 Hippo signaling pathway

chx04392 Hippo signaling pathwaymultiple species

**TABLE S 2**: KEGG PATHWAYS OF GENES IN THE ROH ISLANDS FOR FREE STATE AND GAUTENG POPULATIONS (Continued)

**KEGG ID KEGG Pathways**

chx04510 Focal adhesion

chx04520 Adherens junction

chx04530 Tight junction

chx04540 Gap junction

chx04550 Signaling pathways regulating pluripotency of stem cells

chx04611 Platelet activation

chx04612 Antigen processing and presentation

chx04620 Toll-like receptor signaling pathway

chx04621 NOD-like receptor signaling pathway

chx04622 RIG-I-like receptor signaling pathway

chx04630 JAK-STAT signaling pathway

chx04640 Hematopoietic cell lineage

chx04650 Natural killer cell mediated cytotoxicity

chx04657 IL-17 signaling pathway

chx04658 Th1 and Th2 cell differentiation

chx04659 Th17 cell differentiation

chx04660 T cell receptor signaling pathway

chx04662 B cell receptor signaling pathway

chx04664 Fc epsilon RI signaling pathway

chx04666 Fc gamma R-mediated phagocytosis

chx04668 TNF signaling pathway

chx04670 Leukocyte transendothelial migration

chx04714 Thermogenesis

chx04720 Long-term potentiation

chx04721 Synaptic vesicle cycle

chx04722 Neurotrophin signaling pathway

chx04723 Retrograde endocannabinoid signaling

chx04724 Glutamatergic synapse

chx04726 Serotonergic synapse

chx04727 GABAergic synapse

chx04728 Dopaminergic synapse

chx04730 Long-term depression

chx04740 Olfactory transduction

chx04742 Taste transduction

chx04744 Phototransduction

chx04750 Inflammatory mediator regulation of TRP channels

chx04810 Regulation of actin cytoskeleton

chx04910 Insulin signaling pathway

chx04911 Insulin secretion

chx04912 GnRH signaling pathway

chx04914 Progesterone-mediated oocyte maturation

chx04915 Estrogen signaling pathway

chx04916 Melanogenesis

chx04917 Prolactin signaling pathway

chx04918 Thyroid hormone synthesis

**TABLE S 2**: KEGG PATHWAYS OF GENES IN THE ROH ISLANDS FOR FREE STATE AND GAUTENG POPULATIONS (Continued)

**KEGG ID KEGG Pathways**

chx04919 Thyroid hormone signaling pathway

chx04920 Adipocytokine signaling pathway

chx04921 Oxytocin signaling pathway

chx04922 Glucagon signaling pathway

chx04923 Regulation of lipolysis in adipocytes

chx04924 Renin secretion

chx04925 Aldosterone synthesis and secretion

chx04926 Relaxin signaling pathway

chx04928 Parathyroid hormone synthesis

chx04929 GnRH secretion

chx04930 Type II diabetes mellitus

chx04931 Insulin resistance

chx04932 Non-alcoholic fatty liver disease (NAFLD)

chx04933 AGE-RAGE signaling pathway in diabetic complications

chx04935 Growth hormone synthesis

chx04940 Type I diabetes mellitus

chx04960 Aldosterone-regulated sodium reabsorption

chx04961 Endocrine and other factor-regulated calcium reabsorption

chx04962 Vasopressin-regulated water reabsorption

chx04970 Salivary secretion

chx04971 Gastric acid secretion

chx04972 Pancreatic secretion

chx04974 Protein digestion and absorption

chx04975 Fat digestion and absorption

chx04976 Bile secretion

chx04977 Vitamin digestion and absorption

chx04978 Mineral absorption

chx05010 Alzheimer disease

chx05012 Parkinson disease

chx05014 Amyotrophic lateral sclerosis (ALS)

chx05016 Huntington disease

chx05017 Spinocerebellar ataxia

chx05020 Prion diseases

chx05031 Amphetamine addiction

chx05032 Morphine addiction

chx05033 Nicotine addiction

chx05034 Alcoholism

chx05100 Bacterial invasion of epithelial cells

chx05132 Salmonella infection

chx05133 Pertussis

chx05134 Legionellosis

chx05135 Yersinia infection

chx05140 Leishmaniasis

chx05143 African trypanosomiasis

chx05144 Malaria

**TABLE S 2**: KEGG PATHWAYS OF GENES IN THE ROH ISLANDS FOR FREE STATE AND GAUTENG POPULATIONS (Continued)

**KEGG ID KEGG Pathways**

chx05145 Toxoplasmosis

chx05146 Amoebiasis

chx05150 Staphylococcus aureus infection

chx05152 Tuberculosis

chx05160 Hepatitis

chx05161 Hepatitis B

chx05162 Measles

chx05163 Human cytomegalovirus infection

chx05164 Influenza A

chx05165 Human papillomavirus infection

chx05166 Human T-cell leukemia virus 1 infection

chx05167 Kaposi sarcoma-associated herpesvirus infection

chx05168 Herpes simplex virus 1 infection

chx05169 Epstein-Barr virus infection

chx05170 Human immunodeficiency virus 1 infection

chx05200 Pathways in cancer

chx05202 Transcriptional misregulation in cancer

chx05203 Viral carcinogenesis

chx05205 Proteoglycans in cancer

chx05206 MicroRNAs in cancer

chx05211 Renal cell carcinoma

chx05212 Pancreatic cancer

chx05213 Endometrial cancer

chx05214 Glioma

chx05215 Prostate cancer

chx05216 Thyroid cancer

chx05217 Basal cell carcinoma

chx05218 Melanoma

chx05219 Bladder cancer

chx05221 Acute myeloid leukemia

chx05223 Non-small cell lung cancer

chx05224 Breast cancer

chx05225 Hepatocellular carcinoma

chx05226 Gastric cancer

chx05235 PD-L1 expression and PD-1 checkpoint pathway in cancer

chx05321 Inflammatory bowel disease (IBD)

chx05322 Systemic lupus erythematosus

chx05323 Rheumatoid arthritis

chx05332 Graft-versus-host disease

chx05340 Primary immunodeficiency

chx05412 Arrhythmogenic right ventricular cardiomyopathy (ARV

chx05416 Viral myocarditis

chx05418 Fluid shear stress and atherosclerosis
